# CRISPRO: identification of functional protein coding sequences based on genome editing dense mutagenesis

**DOI:** 10.1186/s13059-018-1563-5

**Published:** 2018-10-19

**Authors:** Vivien A. C. Schoonenberg, Mitchel A. Cole, Qiuming Yao, Claudio Macias-Treviño, Falak Sher, Patrick G. Schupp, Matthew C. Canver, Takahiro Maeda, Luca Pinello, Daniel E. Bauer

**Affiliations:** 1 Division of Hematology/Oncology, Boston Children’s Hospital, Dana-Farber Cancer Institute, Harvard Stem Cell Institute, Broad Institute, Harvard Medical School, Boston, MA 02115 USA; 2 Faculty of Science, Radboud University, 6525 AJ Nijmegen, the Netherlands; 3 Molecular Pathology Unit & Center for Cancer Research, Massachusetts General Hospital and Harvard Medical School, Boston, MA 02114 USA; 4 Center for Cellular and Molecular Medicine, Kyushu University Hospital, Fukuoka, 812-8582 Japan

**Keywords:** CRISPR screen, Protein structure, Tiling, Saturating, Comprehensive, Mutagenesis, Visualization, Software, Domains

## Abstract

CRISPR/Cas9 pooled screening permits parallel evaluation of comprehensive guide RNA libraries to systematically perturb protein coding sequences in situ and correlate with functional readouts. For the analysis and visualization of the resulting datasets, we develop CRISPRO, a computational pipeline that maps functional scores associated with guide RNAs to genomes, transcripts, and protein coordinates and structures. No currently available tool has similar functionality. The ensuing genotype-phenotype linear and three-dimensional maps raise hypotheses about structure-function relationships at discrete protein regions. Machine learning based on CRISPRO features improves prediction of guide RNA efficacy. The CRISPRO tool is freely available at gitlab.com/bauerlab/crispro.

**Electronic supplementary material:**

The online version of this article (10.1186/s13059-018-1563-5) contains supplementary material, which is available to authorized users.

## Background

Clustered regularly interspaced short palindromic repeats (CRISPR) - Cas9 genome editing technologies permit new approaches for the dissection of gene function. Cas9 cleavage results in imprecise end-joining repair products with indels. Biallelic frameshift mutations lead to loss-of-function of the gene product, often through nonsense-mediated decay (NMD) destabilizing the transcript. This paradigm allows for the systematic dissection of genetic dependencies in genome-wide CRISPR screens in the context of disease-relevant cellular phenotypes [1–3]. The mechanisms by which individual alleles contribute to cellular phenotypes are not directly assessed in typical experiments. Such information could aid in the rational design of novel therapeutics as well as in the context of biological engineering to reprogram gene circuitry.

**Fig. 1:**
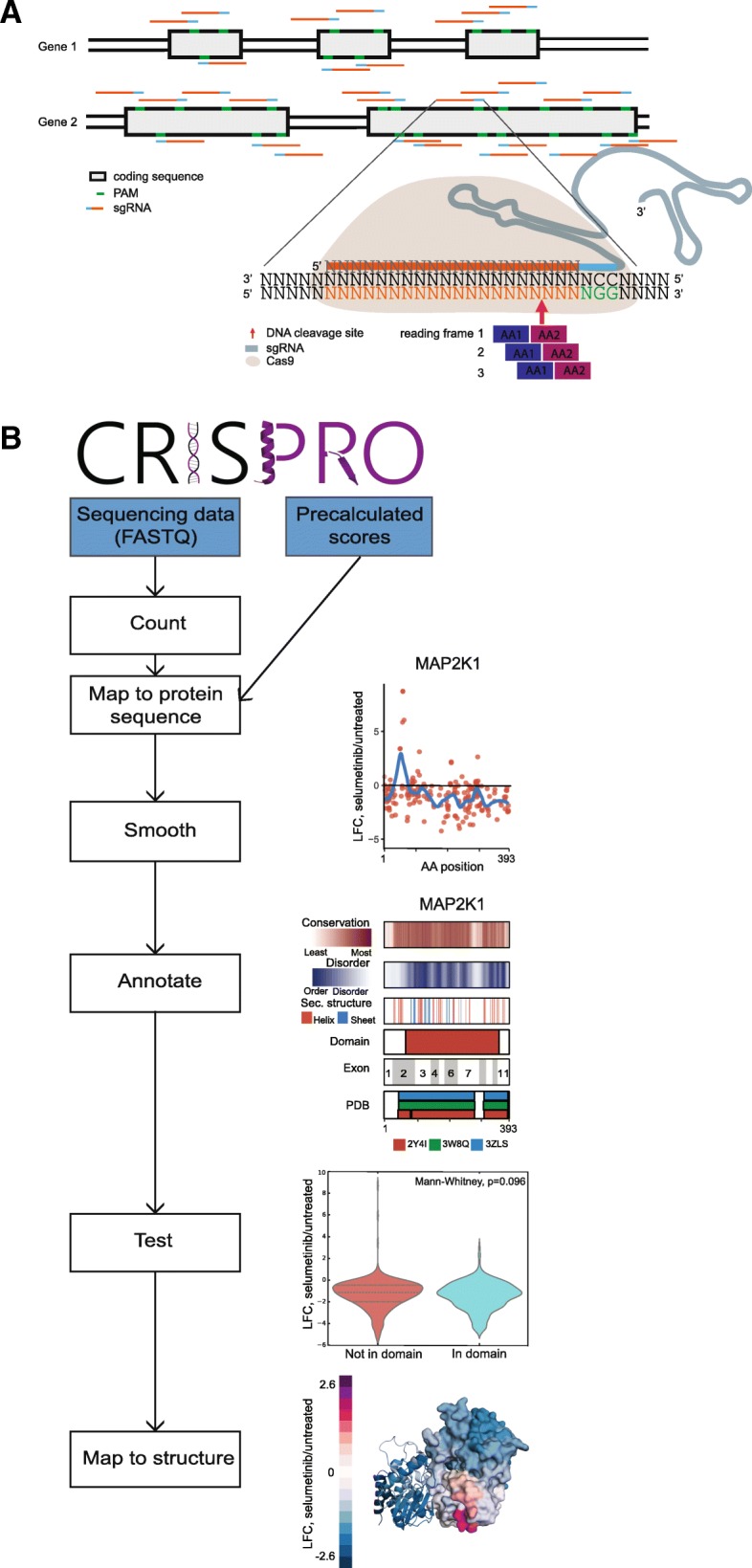
CRISPRO pipeline. **a** Dense mutagenesis of protein coding sequence by pooled CRISPR screening approach. Single guide RNAs target every possible PAM within the coding sequence of a set of genes. Guide RNAs are mapped to the two amino acids closest to the nuclease (e.g., Cas9) cleavage site. **b** Overview of the CRISPRO pipeline. Two input options are either FASTQ files or a precalculated score file (blue). Example data shown for MAP2K1 [8], PDB ID 4MNE

Following a programmable nuclease-mediated double-strand break, the major genome editing outcome is imprecise end-joining, as produced by classical NHEJ and microhomology-mediated end-joining pathways. The ensuing indel spectrum is comprised of short indels, typically up to 10–20 base pair (bp) in length. Although the distribution of indel length is non-uniform and depends on target sequence and cellular repair contexts, on average, 2/3 of alleles from the indel spectrum of end-joining repair following an induced double-strand break (DSB) result in frameshifts. For a gene with two genomic copies and independently assorting repair alleles, on average, ~ 4/9 of edited cells would be expected to produce a biallelic frameshift, causing complete loss-of-function. The remaining ~ 5/9 of cells would retain partial gene function from in-frame alleles, assuming gain or loss of a short stretch of amino acids would be tolerated by the protein. Guide RNAs targeting the coding sequence of critical residues may be associated with heightened functional impact within a population of cells by causing loss-of-function not only from frameshift but also from in-frame mutations [4]. Here, we explore comprehensive dense mutagenesis with many cleavages per gene to systematically define functional protein coding sequences. This method is also known as a CRISPR tiling or guide RNA saturating mutagenesis screen. A typical design would include as many guide RNAs as possible, as restricted by a given protospacer adjacent motif (PAM) availability for a given nuclease (such as the NGG motif in the case of SpCas9) [5, 6]. A single pooled screen experiment may employ large numbers of guide RNAs to systematically disrupt the function of numerous protein-coding genes (Fig. 1a).

Here, we develop a computational tool to gain mechanistic insights into genetic dependencies from dense mutagenesis experiments. We leverage CRISPR tiling screens, protein and nucleotide sequence-level annotations, and 3D visualization of protein structure to elucidate functional residues and predict phenotypic outcome of genome editing in a singular computational pipeline that we have named CRISPRO. To test and develop CRISPRO, we re-analyze previously published data by Munoz et al. [7]. This study describes a set of dense mutagenesis CRISPR screens to investigate the importance of guide RNA positioning in gene inactivation in three different cancer cell lines. We re-analyze CRISPR tiling data from Donovan et al. [8] on *MAP2K1* and *BRAF* as an additional test of CRISPRO. We validate the analytic and predictive power of CRISPRO with prospective dense mutagenesis CRISPR data we generated for *ZBTB7A* and *MYB* [5, 9]. We observe that amino acid sequence conservation, predicted intrinsic protein disorder, and domain structure are highly predictive of the functional requirement of protein sequences. These analyses nominate discrete protein sequences as essential for specific biological phenotypes. We demonstrate the flexibility of the CRISPRO pipeline analyzing orthogonal dense mutagenesis datasets such as ectopic saturation mutagenesis. We derived a machine learning-based model based on CRISPRO features to predict guide RNA efficacy in loss-of-function screens, providing improved predictive performance compared to tools primarily utilizing nucleotide features. The CRISPRO tool is freely available as open-source software along with sample datasets at http://gitlab.com/bauerlab/crispro.

## Results

### Development of the CRISPRO tool

CRISPRO inputs next-generation sequencing datasets resulting from dense mutagenesis CRISPR screens and maps functional scores associated with guide RNAs to genome, transcript, and protein coordinates. We map each guide RNA to the two codons adjacent to the Cas9 cleavage site (see the “Methods” section) (Fig. 1a). The CRISPR scores are smoothed via LOESS regression in order to model local trends of the CRISPR perturbation effect over the entire protein and to provide scores for amino acids with no assigned guides. CRISPRO couples calculation of individual scores for guide RNAs with visualization of functional scores and tracks containing domain structure (InterPro [10]), secondary structure prediction, disordered region prediction, and PROVEAN functional predictions based on interspecies conservation [11–18]. At the tertiary structure level, CRISPRO aligns peptide fragments to existing protein structures in the Protein Data Bank (PDB, www.rcsb.org) and recolors them in a heatmap style reflecting functional scores of amino acid residues [19] (Fig. 1b). These functionally annotated structures may identify critical interfaces between the analyzed protein and other biomolecules as well as inform biophysical and chemical biology hypotheses.

When multiple genes are targeted in a CRISPR screen, CRISPRO defines hit genes with strong functional effect. CRISPRO tests the correlation of hit gene functional scores with annotations. This correlation analysis is conducted for each hit gene individually. In addition, a pooled correlation analysis is conducted for all hit genes together. To test the CRISPRO tool, we evaluated its performance with published datasets. Munoz et al. performed CRISPR pooled screening dense mutagenesis of 139 genes in 3 cancer cell lines [7]. They reported guide RNA sequences with associated log_2_ fold change transformed by *z*-score for cellular dropout. A high dropout score, denoted by a more negative *z*-score, indicates a strong CRISPR phenotype in this study. This data was used as input for CRISPRO. Using default settings, CRISPRO defined 69, 52, and 77 hit genes for the DLD1, NCI-H1299, and RKO cell lines, respectively (at least 75% of guides for a gene having a *z*-score less than 0, see the “Methods” section), largely overlapping the hit genes identified by Munoz et al. (Additional file 1: Figure S1, S9D-E, Additional file 2: Table S1). The default hit calling threshold of CRISPRO is relatively stringent to focus on genes with strong effect sizes and minimize false positive signals. The user can optionally override the CRISPRO default hit gene calling and assign custom hit genes for analysis or avoid hit calling altogether and analyze all genes tested.

CRISPRO can also be used for calculation of functional scores per guide RNA (defined as log_2_ fold change between control and test condition) by using next-generation sequencing (NGS) data as input. The tool includes an option to normalize guide RNA counts to a set of assigned negative control guide RNAs. When using NGS data as input, the tool outputs quality control metrics regarding the deep sequencing data.

### Association of genome editing functional outcome with conservation and disorder

**Fig. 2:**
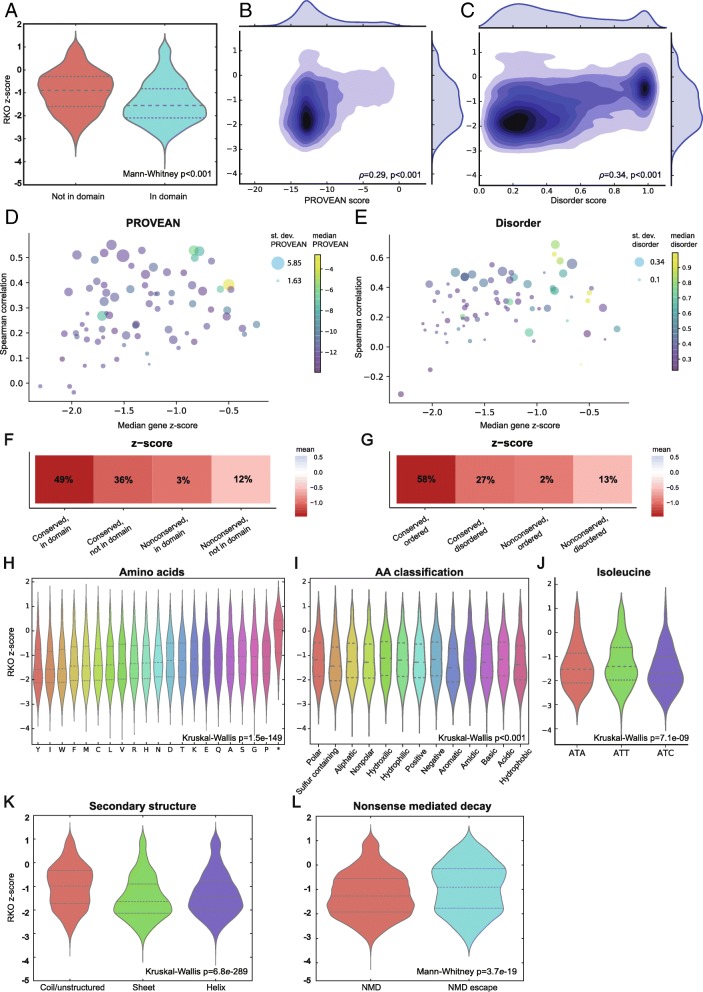
Correlation of annotations to functional scores. Data from Munoz et al. [7] is shown for representative cell line RKO. **a** Violin plot showing the distribution difference for guide RNA RKO *z*-scores targeting inside versus outside of predicted domains (as defined by InterPro). **b** Density plot showing the relation between RKO *z*-score and PROVEAN score (more negative is more conserved). **c** Density plot showing the relation between RKO *z*-score and disorder scores (1 equals disorder, 0 equals order). **d** Scatter plot showing the relation of median RKO *z*-score (*x*-axis), standard deviation (distribution) of PROVEAN score (marker size), and the median of the PROVEAN score (marker color) with the amount of correlation between PROVEAN scores and RKO z-scores (y-axis), for every gene. **e** Analogous to **d**, but for disorder score in place of PROVEAN score. **f** Heatmap showing the mean RKO *z*-score and the percentage guide RNAs falling into groups categorized based on domain annotation and conservation. **g** Heatmap showing the mean RKO *z*-score and the percentage guide RNAs falling into groups categorized based on conservation and disorder score. **h** RKO *z*-score distribution per amino acid. **i** RKO *z*-score distribution per non-mutually exclusive amino acid class: polar (S, T, Y, N, Q); nonpolar (G, A, V, C, P, L, I, M, W, F); hydrophobic (A, V, I, L, M, F, Y, W); hydrophilic (S, T, H, N, Q, E, D, K, R); positively charged (R, H, K); negatively charged (D, E); aliphatic (A, G, I, L, P, V); aromatic (F, W, Y); acidic (D, E); basic (R, H, K); hydroxilic (S, T); sulfur containing (C, M); and amidic (N, Q). **j** RKO *z*-score distribution per codon encoding for isoleucine (I). **k** Distribution of RKO *z*-scores for guides targeting amino acids with different predicted secondary structure: coil/unstructured, sheet, or helix. **l** Distribution for RKO *z*-scores for guides targeting sequences that are predicted to undergo or escape nonsense-mediated decay (NMD)

Targeting amino acids in predicted protein domains is associated with heightened CRISPR functional scores [4, 7]. Using CRISPRO with the Munoz et al. dataset, we can confirm that guide RNAs targeting inside domains show more negative dropout scores than guide RNAs targeting outside a domain (Fig. 2a, Additional file 1: Figure S2A, D, Additional file 3: Table S2). Several groups have previously shown that evolutionary conservation correlates with CRISPR functional scores [7, 20]. We compared the CRISPR functional scores with the PROVEAN conservation scores. For PROVEAN, more negative scores indicate greater conservation. As expected, using the CRISPRO tool, we observed a correlation between conservation and functional scores across all three cell lines tested by Munoz et al. (Spearman correlation, DLD1: *ρ* = 0.24, *p* < 0.001; NCI-H1299: *ρ* = 0.3, *p* < 0.001; RKO: *ρ* = 0.29, *p* < 0.001) (Fig. 2b, Additional file 1: Figure S2B, E). These results are consistent with the hypothesis that targeting conserved as compared to nonconserved protein coding sequences likely gives rise to in-frame loss-of-function alleles. Comparing all the hit genes in the dataset, we observed higher correlation scores between conservation and CRISPR score for genes at which the PROVEAN score have a larger standard deviation. This suggests that PROVEAN scores are most predictive when they are widely distributed for a gene. More conserved genes (lower median PROVEAN score) tended to have a lower median CRISPR score compared to less conserved genes, suggesting that PROVEAN score is not only predictive of the CRISPR score within a gene but also between different genes (Fig. 2d, Additional file 1: Figure S2G,I).

We compared the effects of targeting domain annotated sequences to conserved sequences. We grouped guide RNAs based on both conservation (using PROVEAN score threshold − 6) and domain assignment, resulting in four groups: (1) conserved, in domain; (2) conserved, not in domain; (3) nonconserved, in domain; and (4) nonconserved, not in domain. Comparing the mean of these groups showed that targeting amino acids in a domain and with high conservation has the greatest effect (most negative fitness scores). Within the “not in domain” groups, conserved residues had a more negative mean fitness score than those of nonconserved residues (Fig. 2f, Additional file 1: Figure S2K, M).

We found that protein disorder score was also correlated to functional CRISPR score. Disorder score is a prediction of intrinsically disordered regions (IDRs) within proteins, which also have been called intrinsically unstructured, natively unfolded, natively disordered, or highly flexible regions. Although the classic model posits that “sequence leads to structure leads to function,” IDRs have been found to participate in a wide variety of biological functions of proteins, including interactions with other proteins, nucleic acids, and small molecules, signal transduction, and gene regulation [17, 21, 22]. We hypothesized that given their unstructured nature, IDRs might tolerate short in-frame indels more easily as compared to highly structured regions of proteins. Targeting sequences with higher order (disorder score closer to 0) was associated with enhanced functional scores or higher cellular dropout (Spearman correlation, DLD1: *ρ* = 0.31, *p* < 0.001; NCI-H1299: *ρ* = 0.27, *p* < 0.001; RKO: *ρ* = 0.34, *p* < 0.001) (Fig. 2c, Additional file 1: Figure S2C, F). Similar to the finding for PROVEAN conservation scores, genes with wider distribution of disorder scores (higher standard deviation) demonstrated higher correlation with CRISPR scores compared to those with more narrowly distributed disorder scores. Genes with higher predicted order had higher negative median dropout scores as compared to genes with higher predicted disorder (Fig. 2e, Additional file 1: Figure S2H, J). We tested the relationship between disorder and conservation by grouping guide scores in four categories: (1) conserved, ordered; (2) conserved, disordered; (3) nonconserved, ordered; and (4) nonconserved, disordered (Fig. 2g, Additional file 1: Figure S2L, N). We found the most negative fitness scores for guides targeting conserved and ordered positions. This suggests that conservation and disorder can be used to further refine the set of key functional residues within a protein.

### Association of genome editing functional outcome with protein primary and secondary structure

We evaluated the impact of amino acid identity at the cleavage site by comparing guide RNA dropout scores. Amino acids with largest effect scores across the three cell lines were tyrosine (Y), tryptophan (W), methionine (M), isoleucine (I), and leucine (L) (median scores for these in DLD1 < − 1.25, Kruskal-Wallis: *p* = 3e−136; NCI-H1299 < − 1.7, Kruskal-Wallis: *p* = 1.1e−93; RKO < − 1.39, Kruskal-Wallis: *p* = 1.5e−149) (Fig. 2h, Additional file 1: Figure S3H, J). Selenocysteine (U) also showed a strong effect; however, this rare amino acid was only found twice in the screen and was excluded from further analysis. Tyrosine and tryptophan are the heaviest amino acids (~ 181 and 204 Da), and we hypothesized that their deletion might especially impact protein folding. They are hydrophobic, as are methionine and isoleucine, which may support protein folding [23]. Amino acids were then classified into 13 non-mutually exclusive groups: polar (S, T, Y, N, Q), nonpolar (G, A, V, C, P, L, I, M, W, F), hydrophobic (A, V, I, L, M, F, Y, W), hydrophilic (S, T, H, N, Q, E, D, K, R), positively charged (R, H, K), negatively charged (D, E), aliphatic (A, G, I, L, P, V), aromatic (F, W, Y), acidic (D, E), basic (R, H, K), hydroxilic (S, T), sulfur containing (C, M), and amidic (N, Q). This classification demonstrated more negative CRISPR scores for guide RNAs targeting hydrophobic amino acids as well as the partially overlapping groups of aromatic and sulfur-containing amino acids (Fig. 2i, Additional file 1: Figure S3I, K, S4). We tested if the reason for more negative scores at methionine might be due to targeting the start codon, but methionine at the start position of a protein sequence did not show a significantly different fitness score than methionine throughout the rest of the protein in any of the tested cell lines (Mann-Whitney *U* test, DLD-1: *p* = 0.229; NCI-H1299: *p* = 0.161; RKO: *p* = 0.431) (Additional file 1: Figure S5).

We tested if the impact of disrupting individual codons could be due to the nucleotide identity of the codon itself rather than the encoded amino acid. If the functional effect were solely dependent on the amino acid, different codons for the same amino acid should have a similar score distribution. The only difference in average *z*-score comparing different codons for the same amino acid was observed for isoleucine (Kruskal-Wallis, DLD1: *p* = 6e−13; NCI-H1299: *p* = 9.5e−05; RKO: *p* < 0.001) (Fig. 2j, Additional file 1: Figure S3L, M), where codon ATC had more negative dropout scores than codons ATT and ATA in all three cell lines. Previous data have suggested ATC may have enhanced translation as compared to other codons of isoleucine and may therefore influence protein folding [24, 25].

We predicted a consensus secondary structure by amalgamating the results of several publicly available tools (see the “Methods” section for details). We found that guide RNAs had a greater effect targeting sequences predicted to have helix or sheet secondary structure as compared to coil secondary structure or no secondary structure (Fig. 2k, Additional file 1: Figure S3B, E).

### Association of genome editing functional outcome with mRNA annotations

Nonsense-mediated decay (NMD) is the expected result of the introduction of a premature termination codon (PTC) by a frameshift indel following CRISPR/Cas9 cleavage repair. Exon-junction complex (EJC)-mediated NMD follows the 50 nucleotide rule, meaning that if a PTC resides more than 55 nucleotides upstream of the last exon-exon junction, the terminating ribosome will fail to remove the EJC, causing EJC-mediated NMD. Thus, guide RNAs targeting more than 55 nucleotides upstream of the final exon-exon junction should produce frameshift indels that trigger NMD, whereas guides targeting downstream may produce frameshift indels that escape NMD [26]. We find that when applying this rule, guide RNAs targeting sequences with the ability to escape NMD indeed have less effect on the functional score (Mann-Whitney *U*, DLD1: *p* = 2.2e−37; NCI-H1299: *p* = 1.8e−08; RKO: *p* = 3.7e−19) (Fig. 2l, Additional file 1: Figure S3C, F). These results are consistent with the hypothesis that triggering NMD is a major mechanism of genome editing induced loss-of-function alleles.

We evaluated the predictive value of some other mRNA-level annotations, including propensity for exon skipping, distance to exon-intron junction, and fraction of transcript isoforms targeted. Besides alternative splicing, both point mutations and CRISPR-induced indels can cause exon skipping [27]. We hypothesized that exons that were multiples of 3 would be less functionally essential as compared to those that were not multiples of 3, since mutations could induce exon-skipping and produce mRNA with intact reading frame [28]. We were not able to observe a pervasive impact of exon skipping on CRISPR score, with no significant difference in dropout phenotypes between guide RNAs targeting multiple-of-3 as compared to other exons (Additional file 1: Figure S3A, D, G). We hypothesized that cleavage sites adjacent to exon-intron borders might have heightened functional scores since they could perturb splice sites in addition to protein-coding sequences. However, we were unable to detect a significant difference in guide RNA dropout score for guides targeting close to as compared to distant from exon-intron borders (Additional file 1: Figure S6A, B, D, E, G, H). We hypothesized that targeting sequences shared among transcript isoforms would be more effective than targeting unique isoforms. We observed that the fraction of targeted transcripts only makes a modest difference in CRISPR scores (Spearman correlation, DLD1: *ρ* = 0.068, *p* < 0.001; NCI-H1299: *ρ* = 0.054, *p* < 0.001; RKO: *ρ* = 0.084, *p* < 0.001) (Additional file 1: Figure S6C, F, I).

### Association of genome editing functional outcome with nucleotide annotations

Several tools exist to predict the on-target activity of guide RNAs, which can be defined as the likelihood of creating an indel at a given locus, such as the Doench (2016, Rule Set 2) score, Moreno-Mateos score, and the Wong score, among others [29]. In case of CRISPR experiments utilizing a U6 promoter to express the guide RNA, the Doench score has been shown to have the best performance among the publicly available on-target predictors [29]. Therefore, we focused on the Doench score in our analyses. The Doench score utilizes nucleotide and spacer features like melting temperature without explicitly including protein level features [28]. For CRISPR scores from the Munoz et al. dataset, we found that the Doench score was correlated with observed CRISPR score (Spearman correlation, DLD1: *ρ* = 0.26, *p* < 0.001; NCI-H1299: *ρ* = 0.25, *p* < 0.001; RKO: *ρ* = 0.18, *p* < 0.001) (Additional file 1: Figure S7A, D, G) [30].

We tested predicted frameshift scores with guide RNA score. We hypothesized that guide RNAs more likely to produce frameshift as compared to in-frame alleles would be associated with a greater effect on phenotypic score. We did not detect any association between the out-of-frame score [31] with the phenotypic CRISPR scores (Additional file 1: Figure S7B, E, H).

### Linear maps of genome editing functional outcomes

**Fig. 3:**
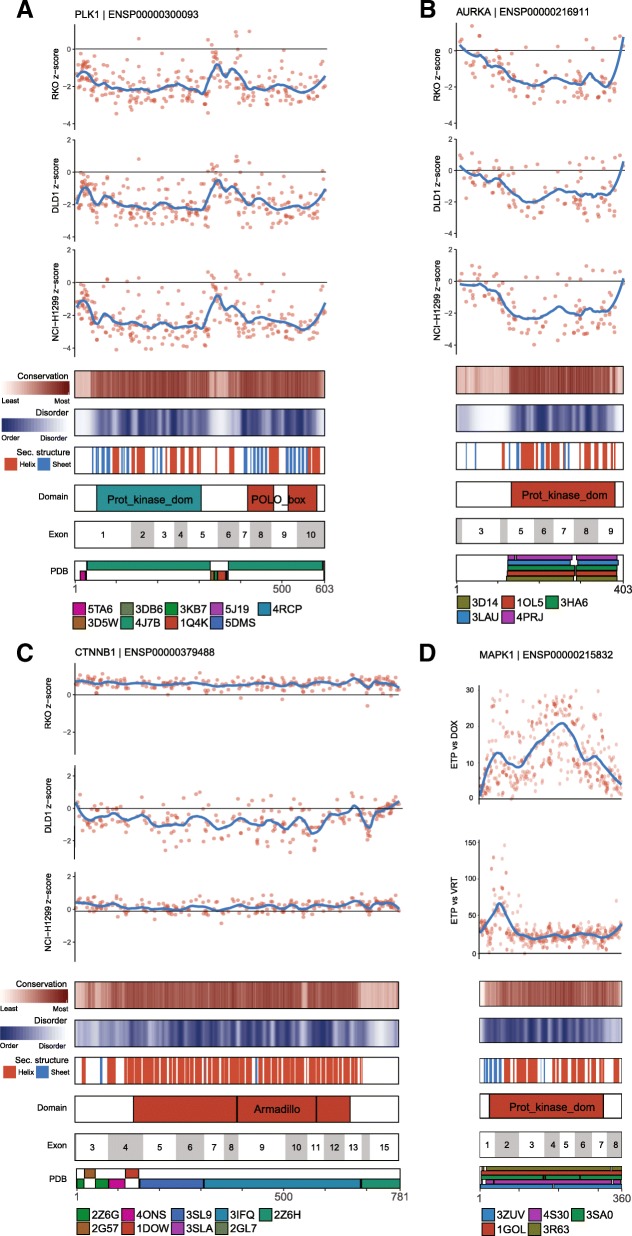
CRISPRO linear maps. **a** *z*-score transformed guide RNA fitness scores for 3 cell lines for *PLK1* from the dataset of Munoz et al. [7] **b** *z*-score transformed guide RNA fitness scores for three cell lines for *AURKA* from the dataset of Munoz et al. [7] **c** *z*-score transformed guide RNA fitness scores for 3 cell lines for *CTNNB* from the dataset of Munoz et al. [7]. **d** *MAPK1*/*ERK2* mutant abundance following DOX induction, relative to early time point (ETP vs. DOX) and *MAPK1/ERK2* mutant abundance following DOX induction in the presence of 3 μM VRT-11E relative to ETP (ETP vs. VRT), from the dataset of Brenan et al. [32]

CRISPRO provides linear tracks to show functional CRISPR scores on a per guide RNA basis. CRISPRO performs LOESS regression on guide RNA functional scores, based on protein primary sequence location. LOESS regression parameters were calibrated by the length of the protein and the assumption that guide RNAs were uniformly distributed throughout a protein (see the “Methods” section). LOESS regression allows interpolation of scores for amino acids that are not targeted by a guide RNA. Several protein-level functional annotations are plotted below the guide RNA scores and LOESS regression, such as PROVEAN conservation scores, disorder scores, secondary structure predictions, InterPro domain annotations [10], and aligned structures available from the PDB. The linear maps are generated for every gene included in the analysis, providing a visual overview of the data and enabling identification of potential regions of interest within a protein at a glance. For example, for *PLK1* and *AURKA* (Fig. 3a, b), the largest negative impact of guide RNAs on cellular fitness is observed at conserved, ordered positions, with secondary structure predictions, and at domains. Reciprocally, the least negative impact on cellular fitness is found at regions with high disorder, little conservation, lack of secondary structure, and without domain annotation. *CTNNB1* (Fig. 3c) is a strong hit gene in only one of the three cell lines tested by Munoz et al., DLD1. In this cell line, there is agreement between the most negative phenotypic CRISPR scores and conservation, disorder, secondary structure, and domain annotation.

The linear mapping functionality of CRISPRO can be readily extended to non-CRISPR datasets. We used CRISPRO to visualize data produced by ectopic saturation mutagenesis of *MAPK1/ERK2* as performed by Brenan et al. [32]. This study tested the function of almost all possible *MAPK1/ERK2* missense mutations to identify gain-of-function and loss-of-function alleles. In the A375 cell line system, loss-of-function *MAPK1* mutants are associated with more rapid proliferation [32]. Following the method of Brenan et al., we summed functional scores for every amino acid substitution at a given position and normalized the summed scores to have a minimal positional score of 0. This resulted in two normalized datasets. One dataset has a normalized score ETP vs DOX, representing the abundance of *MAPK1* mutants following doxycycline (DOX) induction relative to an early time point (ETP) to find loss-of-function alleles. The second dataset has a score ETP vs VRT, presenting the abundance of *MAPK1* mutants in presence of VRT-11E, a small molecule ERK1/2 kinase inhibitor relative to ETP, to find drug-resistance alleles (Fig. 3d). The linear map generated by CRISPRO shows loss-of-function mutants at various sequences with high conservation and low disorder (ETP vs DOX), whereas the drug resistance alleles are concentrated at the ATP-binding pocket around residues 25 to 70 [32] (ETP vs VRT)(Fig. 3d). These data illustrate how CRISPRO can be used to flexibly map a variety of functional scores to protein annotations.

### Visualizing genome editing functional outcomes with protein structures

**Fig. 4:**
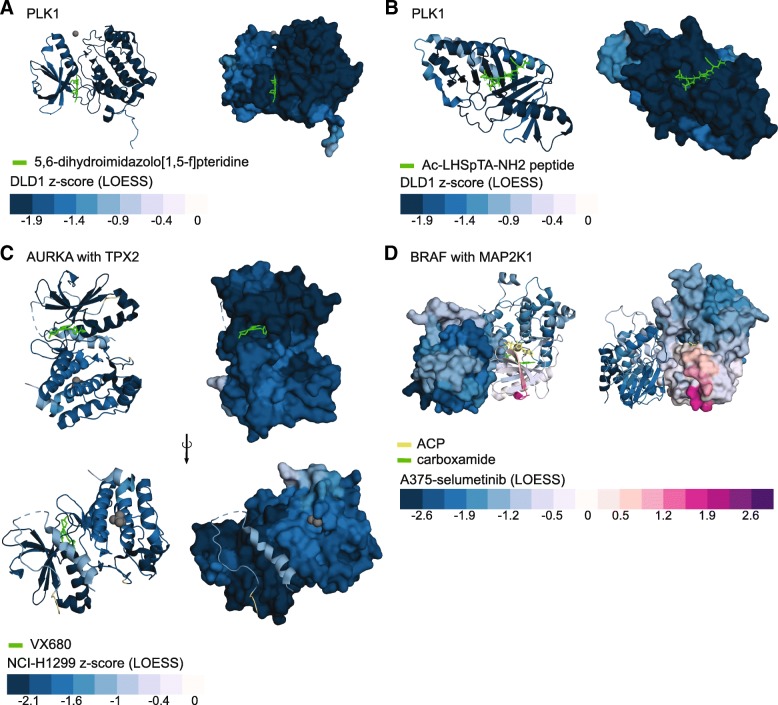
CRISPRO 3D structure maps. **a** PLK1, PDB ID: 5TA6. Mapped scores are DLD1 z-score (LOESS interpolation) of PLK1 (protein kinase domain, AA37-330, cartoon presentation in the left panel, surface presentation in the right panel) in complex with 5,6-dihydroimidazolo[1,5-f]pteridine inhibitor (green). Zinc ion is displayed as a gray sphere. **b** PLK1, PDB ID 3FVH. Mapped scores are DLD1 *z*-score (LOESS interpolation) of PLK1 (polo box domain, AA368-604) in complex with Ac-LHSpTA-NH2 peptide. Both surface (right) and cartoon (left) presentation shown. **C)** AURKA with TPX2, PDB ID 3E5A. Mapped scores are NCI-H1299 *z*-score (LOESS interpolation) of AURKA (presented as surface in left panels, right as a cartoon, AA125-389, protein kinase domain) and TPX2 (presented solely as cartoon, AAs 6–21, 26–42, Aurora-A binding domain) in complex with VX680, an ATP-competitive small molecule inhibitor. Sulfate ions are displayed as gray spheres. **d** BRAF and MAP2K1, PDB ID 4MNE. Mapped scores A375 selumetinib (LOESS interpolation) of BRAF (surface in left panel, cartoon in right, AAs 449–464, 469–722, protein kinase domain) and MAP2K1 (cartoon in left panel, surface in right, AAs 62–274, 307–382, protein kinase domain). Ligands ACP in yellow, and 7-fluoro-3-[(2-fluoro-4-iodophenyl)amino]-*N*-{[(2S)-2-hydroxypropyl]oxy}furo[3,2-c]pyridine-2-carboxamide in green. Magnesium ion is displayed as a gray sphere

To further develop structure-function hypotheses from dense mutagenesis data, CRISPRO maps calculated functional scores to three-dimensional protein structures (Fig. 4). CRISPRO uses BLAST [33] to search the Protein Data Bank (PDB) for all available protein structures and optionally downloads additional structures defined by the user. CRISPRO aligns the structures to the protein sequence and uses PyMOL (The PyMOL Molecular Graphics System. Schrödinger, LCC.) to recolor the structure based on CRISPR scores (see the “Methods” section). By default, CRISPRO sets a two-color heatmap based on the distribution of scores in the dataset such that the more extreme of the 5%ile or 95%ile guide RNA score demarks the last bin and the heatmap is centered around 0 (Additional file 1: Figure S8). Within the Munoz et al. dataset, we observe the lowest fitness scores for *PLK1* in the protein kinase and polo box domains. We mapped interpolated CRISPR scores onto existing protein structures of these domains (PDB IDs 5TA6, 3FVH). The protein kinase domain structure 5TA6 shows the competitive inhibitor 5,6-dihydroimidazolo[1,5-f]pteridine binding at the ATP-binding pocket [34]. The noncatalytic polo box domain structure 3FVH shows the phosphothreonine mimetic peptide Ac-LHSpTA-NH2 binding at a key protein-protein interaction site [35]. Extremely low fitness scores were observed adjacent to these ligand binding sites, demonstrating the capacity of CRISPRO 3D mapping to highlight important protein regions (Fig. 4a, b).

Another example shows the utility of this CRISPRO feature to highlight regions of small molecule interactions as well as protein-protein interactions. *AURKA* is a member of a family of kinases that control progression through mitotic cell division [36]. Figure 4c shows the structure of AURKA in complex with TPX2, a protein that serves as an allosteric activator of AURKA, and VX680, an ATP-competitive small molecule inhibitor of kinase activity (PDB ID 3E5A). Both the interaction sites of AURKA with TPX2 and AURKA with VX680 show extremely low fitness scores (Fig. 4c, Additional file 1: Figure S9). These results demonstrate how CRISPRO analyses and visualization can indicate functional regions of a protein and suggest CRISPRO could help prioritize regions of interest for further chemical biology investigation.

We used CRISPRO to map the results of a CRISPR screen of *MAP2K1* and *BRAF* to available protein structure. This screen was performed in presence of MEK inhibitor selumetinib, to identify drug-resistance alleles [8] (Additional file 1: Figure S9B, C). A positive CRISPR score in the screen indicates an enrichment of these mutants, thus a proliferative effect (drug resistance). A negative CRISPR score means a negative fitness effect, a depletion of these mutants in the cell population (drug sensitivity). The screen was performed in two cell lines, MELJUSO and A375.

No structures of MAP2K1 with selumetinib were available, but the structure PDB ID 4MNE shows the allosteric inhibitors ACP and carboxamide which are thought to occupy the same binding pocket as selumetinib (Fig. 4d). The positive CRISPR phenotypic scores, indicating position of drug-resistance alleles (mapped in purple), showed that these positions are adjacent to the site of small molecule inhibitor binding. Other regions of MAP2K1 distant from small molecule binding only showed negative phenotypic scores, consistent with negative fitness effect from *MAP2K1* loss-of-function. BRAF, which does not directly bind to the small molecule inhibitors, only showed negative fitness scores, with some of the most negative scores concentrated at the BRAF:MAP2K1 protein-protein interaction interface. Overall, these results demonstrate the capacity of the mapping function of CRISPRO to identify critical protein interfaces for functional small molecule active site or allosteric interactions, or sites of protein-protein interactions.

### Prediction of genome editing functional outcome

Given that various CRISPRO features such as conservation and disorder scores were correlated with CRISPR scores, we sought to test if the collection of features and annotations used in CRISPRO could be used to predict guide RNA efficacy in phenotypic screens. Gradient boosting decision tree (GBDT) modeling is one of the current state of the art methods for classification and regression and allows for measurement of feature importance [37, 38]. We initially trained a GBDT model using the Munoz et al. dataset [7], including 10398 sgRNAs targeting 43 genes. For training, the model utilized sgRNA spacer, mRNA, and protein level features as inputs and gene scaled CRISPR scores as the target variable (see the “Methods” section). Performance was measured by calculating the Spearman correlation coefficient between the observed and predicted scaled CRISPR scores for individual genes. We tested the model by 10-fold cross-validation withholding sgRNAs from 10% of genes for testing (to have truly independent sets all the sgRNAs for a gene were withheld if the gene was used in the test set). In addition to GBDT, we compared four regression models for CRISPR score prediction: Lasso, Ridge, Support Vector, and Random Forest. We found similar performance for many of these models, with the GBDT model showing the highest average Spearman correlation coefficient per gene with an average *ρ* = 0.57 (Additional file 1: Figure S10A). Therefore, we focused on GBDT models for further analyses.

We tested the performance of the GBDT model trained on the Munoz et al. data on another saturating mutagenesis dataset, from Doench et al. [30] including 4275 sgRNAs targeting 15 genes. We found that the model showed a substantially lower average Spearman correlation per gene with an average *ρ* = 0.28 (Additional file 1: Figure S10B). Unlike its performance on the Munoz et al. dataset, the GBDT model underperformed the Doench score, which itself was partially derived from analysis of the Doench et al. saturating mutagenesis dataset. We were not surprised that a model trained on a single dataset might be relatively overfitted to that dataset with limited generalizability. To test if the GBDT would be well powered when using the Doench et al. dataset, we re-trained the GBDT model using only this dataset. We observed substantially improved performance, with average Spearman correlation per gene *ρ* = 0.60. As expected, we also observed reciprocally poorer performance for this new model (average Spearman correlation per gene *ρ* = 0.33) when tested on the Munoz et al. dataset (Additional file 1: Figure S10B). This suggested that the two models may capture different properties of those two screens. We reviewed the top features for the GBDT models trained on either the Munoz et al. or Doench et al. datasets (Additional file 1: Figure S10C-D). We indeed observed that different features were assigned relative importance, for example emphasizing PROVEAN score from the Munoz et al. training set and gene fraction from the Doench et al. training set, indicating orthogonal important feature sets learned from the two datasets. Based on these observations we elected to use both datasets for combined training of the GBDT model (Additional file 1: Figure S10B).

**Fig. 5:**
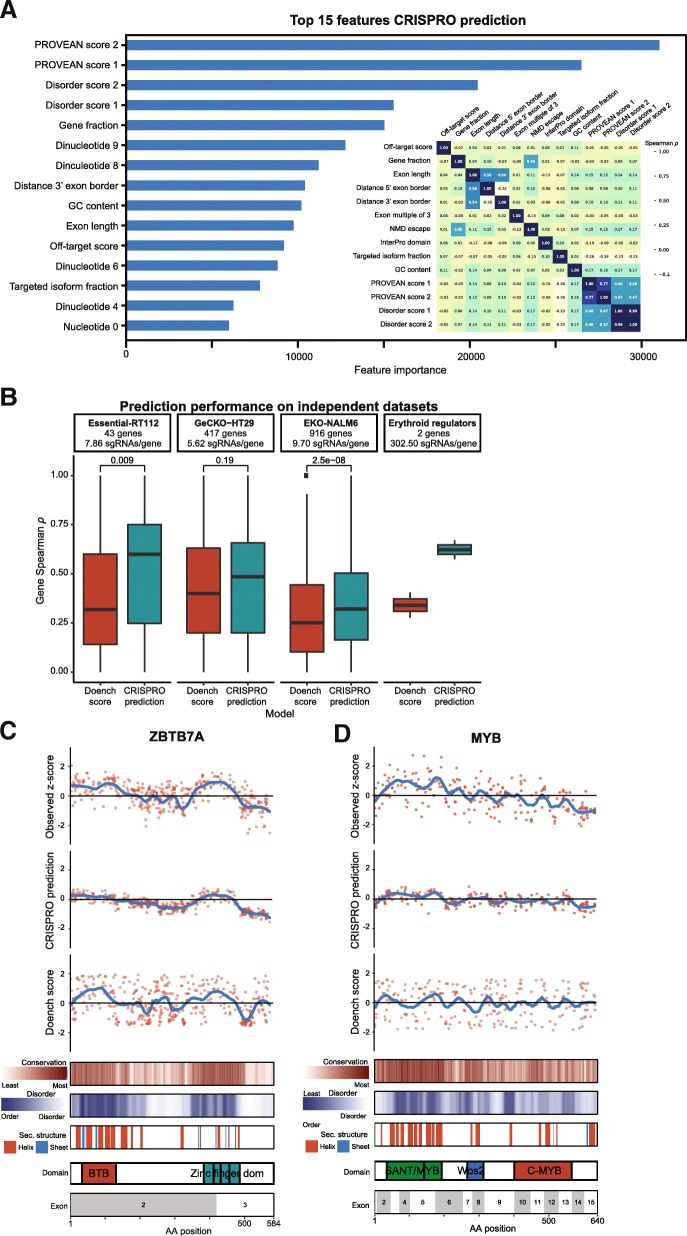
CRISPR score prediction performance on independent datasets. **a** Feature importance in CRISPRO prediction GBDT model by information gain when a feature is used to split the combined training data (Munoz et al. and Doench et al. datasets). Positional nucleotide features are 0-indexed (i.e., nucleotide 0 is in position 1 of the spacer sequence, dinucleotide 0 corresponds to positions 1 and 2 of spacer, where position 20 is PAM proximal). Inset shows pairwise Spearman correlation coefficient for all numerical and binary features in CRISPRO training set. **b** Spearman correlation per gene of predicted as compared to observed CRISPR functional scores in independent datasets not observed in training for Doench score and CRISPRO prediction GBDT model. **c**, **d** Scatter plots for ZBTB7A and MYB of scaled observed guide RNA scores, CRISPRO prediction scores, and Doench scores, with LOESS regression shown by blue lines compared to position in protein. Protein-level and mRNA-level annotations aligned underneath

The most important features by information gain (see the “Methods” section) of the combined training set GBDT model, heretofore called the CRISPRO prediction, were the PROVEAN and disorder scores, followed by relative position targeted in protein (gene fraction), dinucleotides 9 and 8, and distance between predicted double strand break and 3′ exon border (distance 3′ exon border), and GC content of the sgRNA spacer (Fig. 5a, Additional file 1: Figure S11). PROVEAN score and disorder score were modestly correlated, while many of the other features showed low correlation (Fig. 5a inset). This diversity and variable interrelationship of features highlight the complexity of sgRNA efficacy prediction as features apparently affecting Cas9 cleavage and DNA repair (e.g., GC content and nucleotide features), stability of the mRNA gene product (e.g., distance 3′ exon border), and structure-function of the protein gene product (e.g., PROVEAN and disorder scores), all contribute to the CRISPRO prediction model. Given the multiple layers of regulation, we would expect improved predictive performance as more saturating mutagenesis experiments become publicly available and better prognostication of genome editing allelic outcomes emerges. In addition, the prediction is likely influenced by the cell type and biological phenotype measured.

We evaluated the CRISPRO prediction on independent external datasets. These datasets included a CRISPR knockout screen focused on essential genes, Essential-RT112 (43 genes, 7.86 sgRNA/gene) [39], as well as the hit genes from two genome-wide CRISPR knockout screens, GeCKO-HT29 (417 genes, 5.62 sgRNA/gene) [30] and EKO-NALM6 (916 genes; 9.70 sgRNA/gene) [40]. Performance was measured only for genes not observed in training. In each case the CRISPRO prediction provided a higher median and overall a boost over the Doench score in terms of average Spearman *ρ* per gene, significant by Mann-Whitney test in two of these three datasets (Fig. 5b). We observed better generalizability on these unobserved independent test datasets of the CRISPRO prediction model trained on both the Munoz et al. and Doench et al. datasets as compared to models trained on a single dataset (Additional file 1: Figure S10B).

Finally, we performed a prospective saturating mutagenesis experiment, tiling guides throughout the coding sequences of *MYB* and *ZBTB7A*, two key erythroid transcription factors, to test for fitness effects of guide RNAs during erythroid differentiation of a Cas9 expressing human erythroid cell line. For these prospective CRISPR saturating mutagenesis screens, the CRISPRO prediction had substantially higher Spearman correlation coefficient than the Doench score (*ρ* = 0.57 vs 0.28 for MYB; *ρ* = 0.67 vs 0.40 for ZBTB7A) (Fig. 5b). From visual inspection of the linear maps, the CRISPRO prediction accurately identified key functional domains, including the SANT/MYB domains for MYB and the zinc finger domains for ZBTB7A (Fig. 5c, d).

We have calculated CRISPRO prediction scores across the hg19 proteome (available at gitlab.com/bauerlab/crispro). These guide RNA predictions could help genome editing users select for functional studies guide RNAs likely to perturb their gene target.

## Discussion

The discovery of methods for programmable genome editing by CRISPR-Cas9 systems have offered unprecedented capabilities for comprehensive genetic perturbations in situ to investigate the sequence determinants of gene function. We have developed a widely adaptable open-source computational tool, CRISPRO, to take deep sequence data from dense mutagenesis in situ pooled screens as input to compare functional scores with protein, transcript, and nucleotide-level annotations, perform statistical association testing, and visualize functional results with linear maps and three-dimensional protein structures.

We confirmed prior observations that protein-level annotations such as domain structure and interspecies sequence conservation help predict the functional outcome of CRISPR perturbation. Furthermore, we demonstrate that other protein annotations such as disorder score have additional predictive utility.

By automatically mapping the phenotypic scores onto linear and 3D maps, the tool implicates discrete protein regions in specific biological phenotypes. Especially when combined with orthogonal genetic and biochemical data, the ensuing hypotheses may be prospectively tested to improve understanding of protein structure-function relationships and suggest critical interfaces as opportunities for rational targeting for bioengineering or therapeutics.

Beyond protein-level annotations, we observed that transcript level (for example, NMD escape and isoleucine codon usage) and nucleotide level (for example, nucleotide identity) annotations offer additional layers of predictive power. We used these annotations to develop predictive models of genome editing functional outcomes by gradient boosting decision tree modeling. We show boosted performance as compared to prediction by the Doench score alone. We prospectively tested the predictions on orthogonal datasets, and validated the heightened predictive power of the CRISPRO prediction. We have generated prediction scores across all protein coding sequences (available at gitlab.com/bauerlab/crispro).

The CRISPRO tool is flexible to incorporate additional annotations. We anticipate that inclusion of other annotations at various levels, including protein, transcript, chromatin, DNA sequence, and guide RNA, could further increase predictive power and utility of the tool.

A current limitation of Cas9-mediated dense mutagenesis in situ is that the resolution is restricted by both the targeting range constraints of PAM sequence (such as NGG availability for SpCas9) and the variable and difficult to predict end-joining repair indel spectrum following nuclease cleavage dependent on nuclease, guide RNA, and target DNA, chromatin and cellular contexts. However with rapid advances in genome editing technology, the targeting range problem may be partially addressed by use of orthologous and engineered Cas nucleases with alternative PAM restriction, such as the recently described xCas9 and Cas9-NG with NGN PAM [41, 42]. Ability to predict genome editing outcomes may improve with added knowledge of DNA repair determinants and empiric genome editing allele datasets. Furthermore, non-nuclease genomic perturbation options continue to increase, such as the development of C and A base editors [43, 44]. Since the CRISPRO tool is flexible with regard to input data, the resolution of its visualizations and predictive power of its associated annotations will likely only increase as genomic perturbation resolution continues to improve.

Although CRISPRO has been implemented as a tool to aid analysis and prediction of coding sequence perturbations, analogous inclusion of annotations from DNA and chromatin modifications, evolutionary conservation, genetic association studies, and other data types might ultimately be applied to the analysis and prediction of noncoding sequence perturbations as well.

## Conclusions

Here, we describe CRISPRO open-source software for the analysis of dense mutagenesis in situ pooled CRISPR screen datasets. We demonstrate the utility of various protein, transcript, and nucleotide-level annotations to predict functional outcome of genome editing. The linear and 3D maps produced by CRISPRO may be used to develop hypotheses regarding structure-function relationships within mutagenized genes. CRISPRO annotations and models improve prediction of genome editing functional outcome.

## Methods

### CRISPRO pipeline

The CRISPRO pipeline is written completely in Python (The Python Software Foundation, https://www.python.org/) and R [45]. CRISPRO requires Python 2.7 and R > =3.4.1. Packages needed in R are tidyverse (ggplot2, dplyr, lazyeval, gridExtra, purr, RColorBrewer, readr), and DESeq2 (optional, when calculating scores). Package dependencies in Python are pandas (version ≥ 0.21.0), numpy, seaborn, matplotlib (version 1.5.3), PyMOL (version ≥ 2.1.0), scipy, and biopython.

There are two entry points to the CRISPRO pipeline. Users can either upload next-generation sequencing data (sequence read files) in the FASTQ format or scores that have been calculated or precomputed (based on guide RNA or sequence coordinates in combination with the peptide ID).

The overview of the complete pipeline, from input to counting, mapping, annotating, testing and finally displaying the data onto structure, is displayed in Fig. 1b. CRISPRO relies on a precompiled annotation set, which is publicly available for hg19. A script is available to compile other annotation datasets for different genome releases and organisms (e.g., hg38, mm10).

### Counting and mapping guides

The guide RNA counts for a sample are extracted from a given FASTQ file. CRISPRO needs a list of identifiers, sample (condition) names, and comparisons to count the guides in each of the FASTQ files and to calculate functional scores. Identifiers can be either a list of genes, Ensembl peptide, transcript, or gene IDs [46]. Guides are mapped to the protein sequence using information from the CRISPOR database [29]. This database contains all possible guides in the human genome (at coding exons), together with the genomic coordinate where they are predicted to cause a double strand break through Cas9 cleavage. Utilizing the CRISPOR database increases the speed of CRISPRO substantially since the mapping of guides can be precomputed. In addition, users do not have to provide guide sequences to count sequencing output.

CRISPRO maps each guide RNA to the two amino acids nearest the double strand break by using genomic coordinates (Fig. 1a). This avoids the arbitrary decision of mapping a guide to one side of its cleavage site at both the nucleotide and amino acid levels. Also this mapping may more closely approximate the typical indel spectrum following NHEJ repair, affecting 1 up to 10 or more base pairs around the double strand break.

Functional scores are calculated as the log_2_ fold change of the guide count in the sample groups provided and defined by the user. The user has the option to choose if the functional score is calculated by taking the average log_2_ fold change of replicates (ALFC method), or if the log_2_ fold change is calculated by using the DESeq2 R package [47]. CRISPRO uses DESeq2 as a default. DESeq2 shrinks the value of the log_2_ fold change for a guide if read counts are low (noisy), to correct for the higher level of uncertainty. Reducing the fold change allows for confident comparison of all estimated fold changes across experiments.

### Off-target effect

Programmable nuclease mediated genomic cleavages can display modest negative fitness activity, presumably associated with activation of the DNA damage response. Non-targeting guides would not take into account nuclease-mediated gene-independent effects. For this reason, we suggest it is best practice, especially in fitness/growth screens, that scores are normalized to functionally neutral genome targeting guides instead of non-targeting guides [48].

Guide RNAs targeting repetitive genomic sequences can have outsized non-specific negative fitness activity and may confound interpretation of perturbation screens [5]. To avoid high functional CRISPR scores solely caused by a high off-target effect (especially in fitness screens), we suggest it is important to implement an off-target filter. We found in previous data (not shown) guides with a CRISPOR MIT off-target score lower than 5 often have extreme low fitness scores. We included a default filter in CRISPRO to remove any guide RNAs with CRISPOR MIT off-target score less than 5 [29, 49]. This filter can be adjusted by the user.

### Smoothing

Scores for amino acids with no assigned guide RNA are interpolated via LOESS regression in the stats R package, using known guide scores and location to train the model. LOESS regression is nonparametric, and uses weighted least squares to fit a quadratic curve on a contiguous subset of the data, in order to capture local trends of the CRISPR effect over the entire protein. The size of the subset of the data to which to fit a curve is determined by the span parameter, which is defined as 100/protein length for a given protein. The span parameter allows for approximately the same amount of data to be used to fit a local curve for various length genes with the assumption of uniform distribution of guide RNAs. The optimal span parameter should avoid both under-smoothing with excessive variance, and over-smoothing with loss of information. The parameter was set empirically based on correlation between the LOESS regression curve and other protein annotations such as PROVEAN and disorder scores (Additional file 1: Figure S12). We compared span parameters ranging from 10 AA/L to 250 AA/L, where L is the length of the protein in AA, in terms of the correlation of PROVEAN score and disorder score with CRISPRO functional scores (Additional file 1: Figure S12). We observed that the correlation increased sharply as the span was extended from 10 to ~ 50 AA/L, but between ~ 50–250 AA/L there was a relative plateau in the correlation. We chose 100 AA/L as a pragmatic solution to attempt to balance risk of under-smoothing and over-smoothing.

### Annotations

Annotation of sequences and testing their correlation with calculated CRISPR scores is essential to the analysis in the CRISPRO pipeline. Sequences may influence CRISPR scores via effects at the DNA, RNA, or protein levels. At the DNA level, the target sequence and its surrounding context may specify guide RNA binding efficiency, off-target potential, or genomic repair preferences. Edits may affect mRNA splicing (by impacting cis-acting splice regulatory sequences), RNA stability (such as frameshifts that initiate nonsense-mediated decay), or isoform usage (by targeting unique as compared to shared exons). At the protein level, the primary amino acid identity, secondary structures, likelihood of disorder, presence in identified domains, or interspecies/intraspecies constraint may influence the impact of mutations. CRISPRO utilizes one precompiled database with annotations from several genome-wide databases.

Annotations from publicly available databases include CRISPOR (guide efficiency score (Doench ’16 [30]), out-of-frame score and off-target score), InterPro (domains), APPRIS (protein principal isoform), and Ensembl (exons, peptide and coding sequences) [10, 29, 50]. The CRISPRO database also contains precomputed conservation scores (PROVEAN [15]), exon length, DSB distance to 3′ and 5′ exon borders, the location in the protein (protein fraction), the predicted ability to escape nonsense-mediated decay (NMD) (when the guide RNA targets upstream of − 55 bp from the final exon-exon junction), the fraction of targeted protein isoforms per gene, disorder score, and secondary structure prediction.

PROVEAN (Protein Variation Effect Analyzer) is a protein sequence variant predictor that not only predicts the effect of single amino acid substitutions, like other commonly used tools such as PolyPhen and SIFT, but also predicts the effect of deletions. Since CRISPR-Cas9 cleavage creates a spectrum of indels, CRISPRO uses the effect score for single amino acid deletions generated by PROVEAN as a measure of conservation. More negative PROVEAN scores indicate greater conservation. CRISPRO’s original database is designed for hg19 proteins from Ensembl release 90; we computed all PROVEAN scores for this database.

As described above, the DSB coordinate for each guide is obtained from the CRISPOR database. We mapped guide RNAs to their corresponding amino acids in a protein and calculated the distance to both exon borders, based on protein (genomic) coding coordinates from hg 19 Ensembl, release 90 (start and end points per exon).

We pre-computed disorder scores for CRISPRO with VSL2b, a length-dependent predictor [17, 18].

We used multiple tools, PSSpred, PSIpred, SPINE X, and RaptorX, to build a weighted consensus secondary structure prediction [11–14, 16]. Each tool provides a probability score for a predicted secondary structure (either strand (B), helix (H) or coil (C)). For each amino acid, these scores are added up per secondary structure and divided by the sum of all the options. This gives the weighted predictive score per secondary structure, whichever is the highest determines which secondary structure is predicted.

Two BLAST searches are used to align and annotate all available protein structures in the RCSB Protein Data Bank (PDB) [19, 33]. The first search is done with complete protein sequences of the entire genome. These hits and alignments are directly available in CRISPRO’s standard annotation set. The second search is done per protein domain, as defined by the SMART database, to expand the range of available structures and to include partial structure hits which might have been missed in the first round of BLAST. For both BLAST searches the cut-off value for identity is 0.7 and *e*-value is 0.05. The results of the second BLAST search (domain only) are separated in an additional annotation file. These results are only used when a CRISPRO-user includes the option to map functional scores to structures. Any additional structures available for a protein are in that case aligned with Biopython pairwise2 local alignment (using blosum62 matrix, gap open penalty: − 10, gap extension penalty: − 0.5) [51]. The option exists for the user to pass extra PDB IDs (which might not have been found by the automated BLAST search) and the corresponding protein ID as input for CRISPRO. These structures would also be aligned with Biopython pairwise2 (same variables).

### General quality control and statistical testing

As part of its standard output CRISPRO provides summary statistics, quality information, guide density, functional scores and annotations based on raw FASTQ sequencing files. For each FASTQ file used as input, the following is calculated: total reads, mapped reads, percentage mapped reads, Gini score (a measure of inequality of the distribution), mean reads per guide, standard deviation reads per guide, minimum reads per guide, 10th percentile reads per guide, median reads per guide, 90th percentile reads per guide, and maximum reads per guide. All these values contribute to the quality control of the sequencing data and its mapping. Raw read counts per guide are saved for each of the sequencing files (samples) and a Pearson correlation test is performed comparing all sequencing files.

CRISPRO calculates guide density and average guide distance for each gene individually. Guide density is calculated by dividing the total number of guides in a protein by the total number of amino acids. The distance between each of the guides is based on the first amino acid in the sequence it maps to, which is then averaged for all guides in a protein. Guides are filtered based on detection in the sequencing data. In other words, if according to CRISPOR there was a possible guide targeting the protein coding sequence, the guide is only considered if it was actually detected in the sequencing files and has a functional score.

Each guide RNA score is normalized by subtraction of the median negative control guide RNA score (if a set of negative control guides is available). It is optional for the user to assign negative and positive controls as input for CRISPRO. Negative controls can either be nontargeting guides or neutral gene-targeting guides. The latter is encouraged when possible, to control for the expected effect of gene-independent genome targeting events. Positive control guide RNAs could be targeting genes with known high effect, such as guides targeting ribosomal genes in the case of negative selection screens.

CRISPRO calculates the mean, standard deviation, first quartile, median, third quartile, the interquartile range (IQR), and the earth mover’s distance for the functional scores of each tested gene. The earth mover’s distance indicates the cost of turning the distribution of scores of the protein into the distribution of the negative control distribution.

Operationally, CRISPRO defines a gene as a hit for a given score (i.e., showing an overall phenotype of potential biological interest) in the CRISPR screen by checking if at least 75% of guides are above or below 0 (e.g., the IQR does not contain 0), where 0 corresponds to the median of the distribution of the negative controls. If this is the case, the gene is labeled as hit. We have found that performing statistical tests, like Mann-Whitney, between guides targeting a gene and nontargeting controls leads to the classification of most genes as hits. Small effect sizes may be statistically significant, because of the usually high number of tested guides. The tendency to identify many genes as significant hits may be exaggerated with use of nontargeting guides as negative control as compared to neutral genes [48]. For the purpose of further statistical testing, the direction of the hit is assigned, labeling the hit gene as either positive (median > 0) or negative (median ≤ 0). It is possible for the user to define gene hits as an input for CRISPRO, by adding a list of gene names, or Ensembl peptide, transcript or gene IDs. If the user chooses to do so, the default of using the IQR will be overwritten.

CRISPRO generates several plots to show correlation between every annotation CRISPRO provides and the functional scores. For categorical annotations these are violin or box plots, for continuous data these are scatter plots. CRISPRO produces plots for each score for all hit genes pooled and for the individual hit genes. CRISPRO performs relevant statistical tests for each annotation (either Spearman correlation, Mann-Whitney test, or Kruskal-Wallis test with SciPy module in Python [52]).

### Mapping CRISPR scores to protein structures

CRISPRO downloads all structures found by BLAST search in the PDB (as described above), when the user chooses to map functional CRISPR scores to protein structures. In case there are specific structures the user wants to map, regardless if these were found in the standard BLAST search, the user has the option to pass the PDB IDs and the corresponding protein ID as input for CRISPRO. These structures will be included in all other standard output for CRISPRO, like the figures presenting annotations (linear tracks) and overview tables. Every PDB structure found (complying with before mentioned conditions of the BLAST search) or added by the user will be mapped and recolored, even if there are multiple structures available for the same (sub)sequence of a protein.

CRISPRO saves the amino acid sequence of the structure via PyMOL and aligns with the full protein sequence. Based on these alignments, CRISPRO writes raw input text files for PyMOL, containing a list with the CRISPR functional score values corresponding to each amino acid present in the structure. It might occur that a structure has a different sequence than the original protein sequence, in which case there may be mismatches between amino acids, amino acids missing, or extra amino acids in the structure. If there are amino acids in the structure that are different but aligned to an amino acid in the original protein, the corresponding score is mapped. If there are extra amino acids in the structure which cannot be aligned, no data will be mapped (shown in yellow).

CRISPRO loads the functional CRISPR scores in the B-factor field of the PDB structures in PyMOL. To recolor the structure based on these values, CRISPRO assigns a bin and corresponding color to each amino acid in the structure. The standard CRISPRO color legend consists of either 17 or 9 bins, from blue to dark purple, centered on 0. To be able to visually compare proteins and to distinguish important regions, CRISPRO determines bin size and boundaries for each functional CRISPR score (separately for both raw and LOESS regressed scores), over all the proteins in the dataset. Either the 5th or 95th percentile (and its inverse) of the score distribution, whichever is farther from 0, is set as the upper and lower border of the outermost bins. Every score lower or higher than this value will fall into those outer bins. The rest of the bins are evenly sized between the borders, resulting in a scale centered on 0 (Additional file 1: Figure S8).

The recolored structures are saved as PyMOL session files (.pse). The user can open the sessions in the desktop version of PyMOL and adjust the orientation or visuals of the structure before saving an image.

### Score prediction

#### Data processing

For each gene, we multiplied each CRISPR score (average of all guide RNA CRISPR scores) for a gene by − 1 if the mean score of the guide RNAs was less than 0, and *z*-score normalized them. By doing so, a predicted high CRISPR score is interpreted as having the greatest effect on phenotype for that gene, regardless of direction. We then scaled and centered CRISPR scores by gene, to make the target variable comparable across experiments.

#### Models

For Lasso and Ridge Regression, we used LassoCV and RidgeCV respectively from the scikit-learn package in Python with default parameters to determine the optimal alpha parameter via the default cross validation method [53]. SVR from scikit-learn was used for support vector regression model. We used LGBMREgressor, from the LightGBM package in Python, for the GBDT and random forest algorithms described above [54]. We explored the hyperparameter space for the gradient boosted decision trees using GridSearchCV from the scikit-learn package in Python [53], yielding the following parameters differing from the default: (“bagging_freq” 0, “colsample_bytree” 1/3, “learning_rate” 0.01, “max_depth” − 1, “min_child_samples” 32, “n_estimators” 1024, “max_bin” 63.

We performed cross-validation by leaving out guides targeting 10% of genes in the full training set (43 genes).

#### Features

Targeted amino acids 1 and 2, domain occupancy status (InterPro), exon multiple of 3, ability of targeted transcript to escape nonsense-mediated decay, single nucleotide and dinucleotide positional identities within guide RNA spacer (e.g., identity of nucleotide at position 17 in spacer), and orientation of sgRNA relative to gene (e.g., both sgRNA and gene involve same strand) were all used as categorical features. Categorical features were one hot encoded. Numerical features included PROVEAN deletion score of the targeted amino acids 1 and 2, position in the gene, predicted disorder score of amino acids 1 and 2, GC content of the 20-mer guide, length of the targeted exon, and off-target score of the guide RNA. We computed GC content of the 20mer guide by adding the number of observed “G”s and “C”s in the 20mer and dividing the sum by the length of the guide (20 bp).

For Lasso, ridge, and support vector models, the feature set was scaled to have a range of 0–1. Features were removed recursively in 10 group fold cross validation using scikit-learn package in Python [53].

#### Feature importance (GBDT)

Feature importance was calculated via information gain of split with the LightGBM package in Python [54].

#### Training set processing

In the dataset from Munoz et al., each sgRNA had a log_2_ fold change in three cell lines. We used the average log_2_ fold change across the 3 cell lines for each guide (“average score”). Next, we filtered out genes that had a mean “average score” > − 1 (to filter potential outliers that could have biased the model). In the CRISPR saturating mutagenesis from Doench et al., we calculated log_2_ fold change of DMSO day 14 over ETP.

#### Independent test set processing

For each dataset utilized [30, 39, 40], the authors provided a list of genes classified as hits from the respective CRISPR screen. Only sgRNAs from hit genes were utilized for testing. If sgRNA scores were provided for each replicate, the average was used for downstream data processing described above. If normalized counts were provided for a replicate/condition, sgRNA scores were calculated as described in the methods of the corresponding paper. SgRNAs from genes that had sgRNAs observed in testing were removed.

### Saturating mutagenesis CRISPR/Cas9 fitness screen in HUDEP-2

HUDEP-2 cells constitutively expressing lenti-Cas9 were transduced with a lentiviral guide RNA library containing puromycin resistance. 24 h post transduction, cells underwent selection and erythroid based differentiation protocol. After 12 days of culture, we isolated the genomic DNA allowing for next-generation sequencing (NGS) of the integrated guide RNA library as previously described [5]. We defined the fitness score as the log_2_ fold change of counts in the final time point over the counts in the lentiviral plasmid sample.

## Additional files

Additional file 1 Supplemental figures S1-12. (PDF 20535 kb)

Additional file 2: **Table S1**, Hit analysis RKO, DLD1 and NCI-H1299 cell lines (XLSX 74 kb)

Additional file 3: **Table S2**, Annotated CRISPRO output (XLSX 33034 kb)

## Abbreviations

BR: Base pair
CRISPR: Clustered regularly interspaced short palindromic repeats
EJC: Exon-junction complex
ETP: Early time point
GBDT: Gradient boosting decision tree
IDR: Intrinsically disordered region
Indel: Insertion and deletion
LFC: Log_2_ fold change
NGS: Next-generation sequencing
NHEJ: Non-homologous end joining
NMD: Nonsense-mediated decay
PAM: Protospacer adjacent motif
PDB: Protein Data Bank
PDP: Partial dependency contour plot
PTC: Premature termination codon
sgRNA: Single guide RNA
