# Supplementary material for: CRISPRO: identification of functional protein coding sequences based on genome editing dense mutagenesis
Source: Genome Biol. 2018 Oct 19;19:169. doi: 10.1186/s13059-018-1563-5 (PMC6195731; doi:10.1186/s13059-018-1563-5)
Supplement: Supplementary file 1 — Supplemental figures S1-12. (PDF 20535 kb) [file 13059_2018_1563_MOESM1_ESM.pdf]

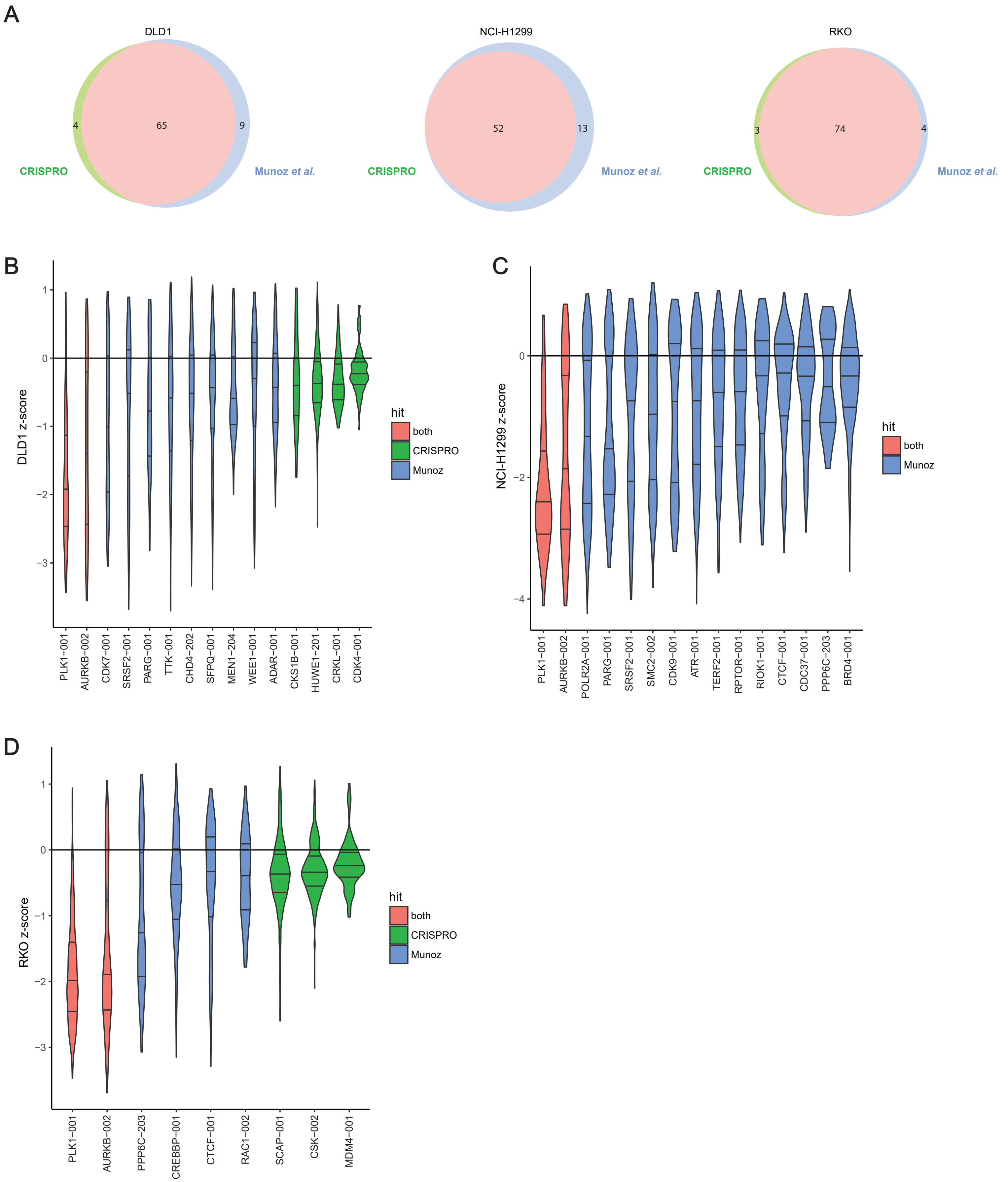

Figure S1 Hit genes.

A) Venn diagrams for each of 3 cell lines, showing assigned number of hit genes per method of CRISPRO and Munoz *et al*.. Munoz et al. defined hits by setting a cut-off at an average z-score of -0.4, defining 74 (DLD1), 65 (NCI-H1299), and 78 (RKO) hits. Out of the hits set by Munoz *et al.*, CRISPRO identified 65, 52, and 74 hits from DLD1, NCI-H1299, and RKO, missing a few hits which had a mean just below the -0.4 cut-off, with a relatively large standard deviation. Hits unique to CRISPRO had average z-scores close to -0.4, but had a smaller distribution of scores (Supplementary Table 1). CRISPRO assigned 4 and 3 additional hits for DLD1 and RKO cell lines. **B-D)** Violin plots for hits uniquely defined by CRISPRO or Munoz *et al*., with two example hits identified by both methods (PLK1 and AURKB) for each cell line: **(B)** DLD1, **(C)** NCI-H1299, **(D)** RKO.

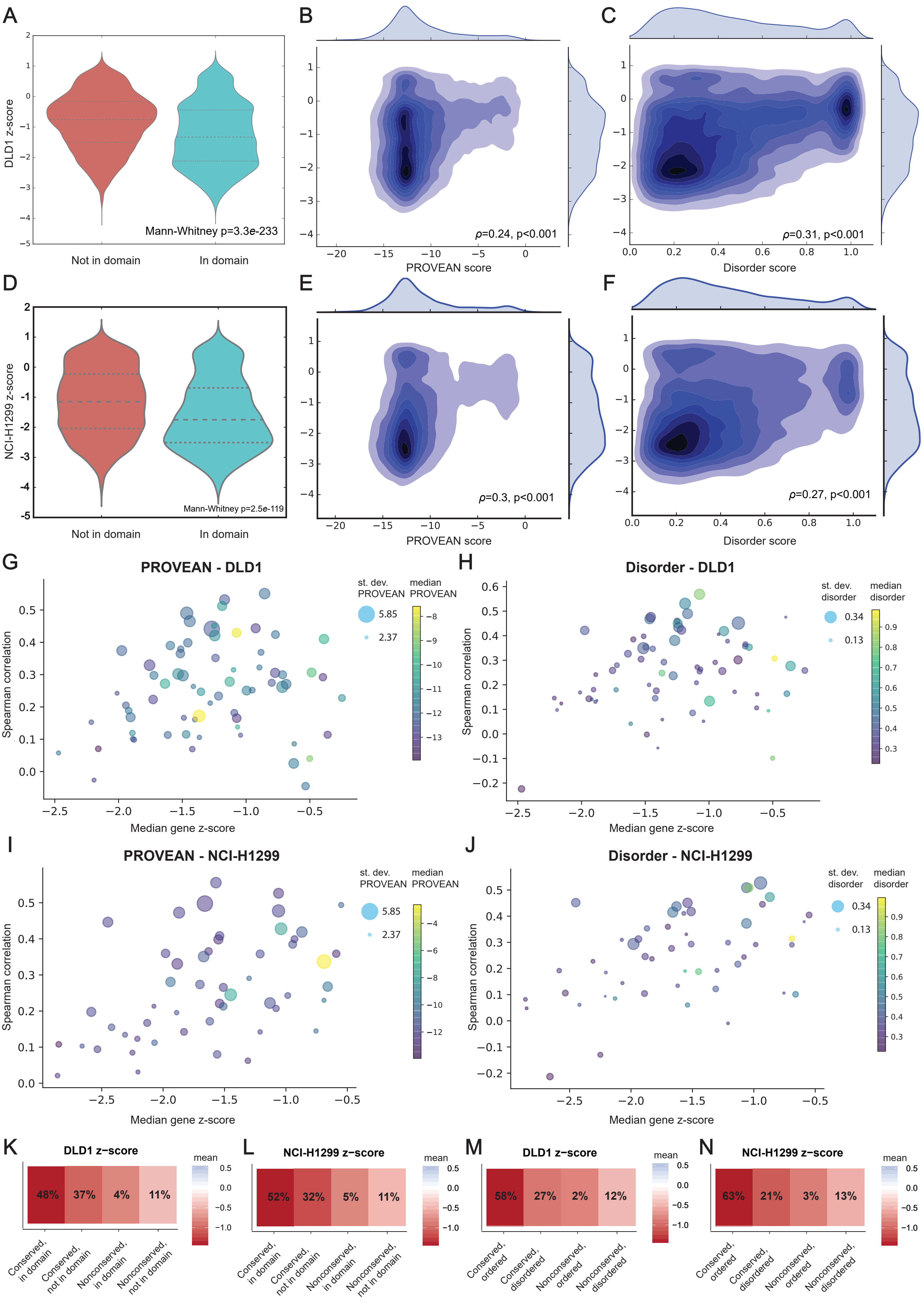
A
B
C
D
E
F
G
H
I
J
K
L
M
N
DLD1 z-score
Mann-Whitney p=3.3e-233
Not in domain
In domain
ρ=0.24, p<0.001
PROVEAN score
ρ=0.31, p<0.001
Disorder score
NCI-H1299 z-score
Mann-Whitney p=2.5e-119
ρ=0.3, p<0.001
ρ=0.27, p<0.001
PROVEAN - DLD1
Disorder - DLD1
PROVEAN - NCI-H1299
Disorder - NCI-H1299
Spearman correlation
Median gene z-score
st. dev. PROVEAN
median PROVEAN
5.85
2.37
st. dev. disorder
median disorder
0.34
0.13
DLD1 z-score
NCI-H1299 z-score
mean
48%
37%
4%
11%
52%
32%
5%
11%
58%
27%
2%
12%
63%
21%
3%
13%
Conserved, in domain
Conserved, not in domain
Nonconserved, in domain
Nonconserved, not in domain
Conserved, ordered
Conserved, disordered
Nonconserved, ordered
Nonconserved, disordered

Figure S2 Correlation of annotations to functional scores for cell lines DLD1 and NCI-H1299, similar to Figure 2.

A) Violin plot showing the distribution difference for guide RNA DLD1 z-scores targeting inside versus outside of predicted domains (as defined by InterPro). **B)** Density plot showing the relation between DLD1 z-score and PROVEAN score (more negative is more conserved). **C)** Density plot showing the relation between DLD1 z-score and disorder scores (1 equals disorder, 0 equals order). **D)** Violin plot showing the distribution difference for guide RNA NCI-H1299 z-scores targeting domains versus targeting outside of predicted domains (as defined by InterPro). **E)** Density plot showing the relation between NCI-H1299 z-score and PROVEAN score (more negative is more conserved). **F)** Density plot showing the relation between NCI-H1299 z-score and disorder scores (1 equals disorder, 0 equals order). **G)** Scatter plot showing the relation of median DLD1 z-score (x-axis), standard deviation (distribution) of PROVEAN score (marker size), and the median of the PROVEAN score (marker color) with the amount of correlation between PROVEAN scores and DLD1 z-scores (y-axis), for every transcript. **H)** See **G**, here for disorder and DLD1 z-score. **I)** see **G**, here for NI-H1299 scores. **J)** See **G**, here for disorder and NCI-H1299 z-scores. **K)** Heatmap showing the mean DLD1 z-score and the percentage guide RNAs falling into groups categorized based on domain annotation and conservation. **L)** See **K**, here for NCI-H1299 z-scores.

M) Heatmap showing the mean DLD1 z-score and the percentage guide RNAs falling into groups categorized based on conservation and disorder score. **N)** See **M**, here for NCI-H1299 z-scores.

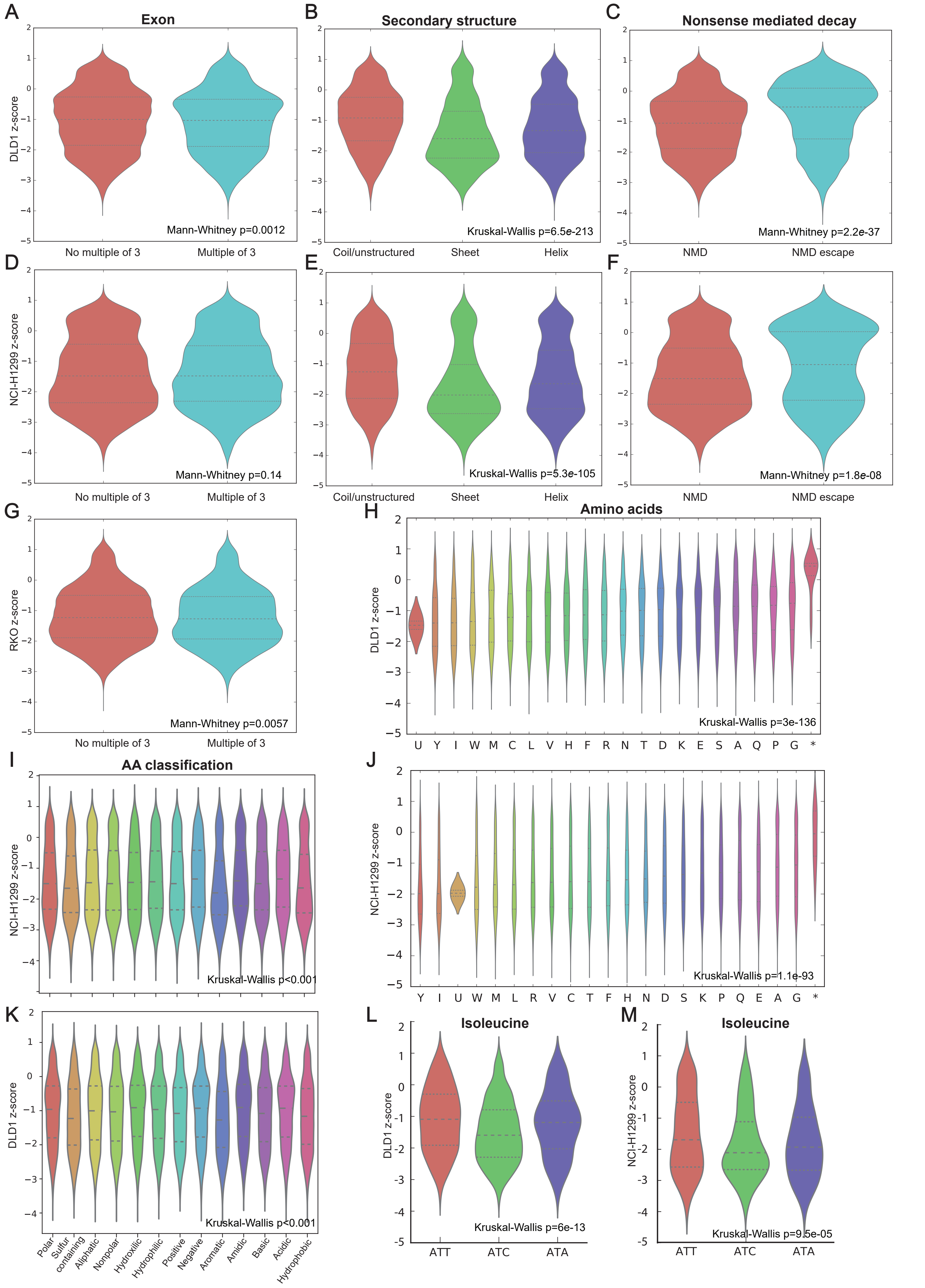

A
Exon
DLD1 z-score
Mann-Whitney p=0.0012
No multiple of 3
Multiple of 3
B
Secondary structure
Kruskal-Wallis p=6.5e-213
Coil/unstructured
Sheet
Helix
C
Nonsense mediated decay
Mann-Whitney p=2.2e-37
NMD
NMD escape
D
NCI-H1299 z-score
Mann-Whitney p=0.14
No multiple of 3
Multiple of 3
E
Kruskal-Wallis p=5.3e-105
Coil/unstructured
Sheet
Helix
F
Mann-Whitney p=1.8e-08
NMD
NMD escape
G
RKO z-score
Mann-Whitney p=0.0057
No multiple of 3
Multiple of 3
H
Amino acids
DLD1 z-score
Kruskal-Wallis p=3e-136
U Y I W M C L V H F R N T D K E S A Q P G *
I
AA classification
NCI-H1299 z-score
Kruskal-Wallis p<0.001
J
NCI-H1299 z-score
Kruskal-Wallis p=1.1e-93
Y I U W M L R V C T F H N D S K P Q E A G *
K
DLD1 z-score
Kruskal-Wallis p<0.001
Polar
Sulfur containing
Aliphatic
Nonpolar
Hydroxilic
Hydrophilic
Positive
Negative
Aromatic
Amidic
Basic
Acidic
Hydrophobic
L
Isoleucine
DLD1 z-score
Kruskal-Wallis p=6e-13
ATT
ATC
ATA
M
Isoleucine
NCI-H1299 z-score
Kruskal-Wallis p=9.5e-05
ATT
ATC
ATA

Figure S3 Correlation of transcript and protein level annotations to functional scores.

A) Distribution of DLD1 z-scores for guide RNAs targeting in exons that are a multiple of 3 or not.

B) Distribution of DLD1 z-scores for guide RNAs targeting amino acids with different predicted secondary structure: coil/unstructured, sheet, or helix. **C)** Distribution for RKO z-scores for guide RNAs predicted to trigger or escape NMD. **D)** Distribution of NCI-H1299 z-scores for guide RNAs targeting in exons that are a multiple of 3 or not. **E)** Distribution of NCI-H1299 z-scores for guide RNAs targeting amino acids with different predicted secondary structure: coil/unstructured, sheet, or helix. **F)** Distribution for NCI-H1299 z-scores for guide RNAs predicted to trigger or escape NMD.

G) Distribution of RKO z-scores for guide RNAs targeting in exons that are a multiple of 3 or not.

H) DLD1 z-score distribution per amino acid. **I)** DLD1 z-score distribution per non-mutually exclusive amino acid class: polar (S, T, Y, N, Q); nonpolar (G, A, V, C, P, L, I, M, W, F); hydrophobic (A, V, I, L, M, F, Y, W); hydrophilic (S, T, H, N, Q, E, D, K, R); positively charged (R, H, K); negatively charged (D, E); aliphatic (A, G, I, L, P, V); aromatic (F, W, Y); acidic (D, E); basic (R, H, K); hydroxilic (S, T); sulfur containing (C, M); and amidic (N, Q). **J)** NCI-H1299 z-score distribution per amino acid. **K)** NCI-H1299 z-score distribution per amino acid class, similar to **I**. **L)** DLD1 z-score distribution per codon encoding for isoleucine (I). **M)** NCI-H1299 z-score distribution per codon encoding for isoleucine (I).

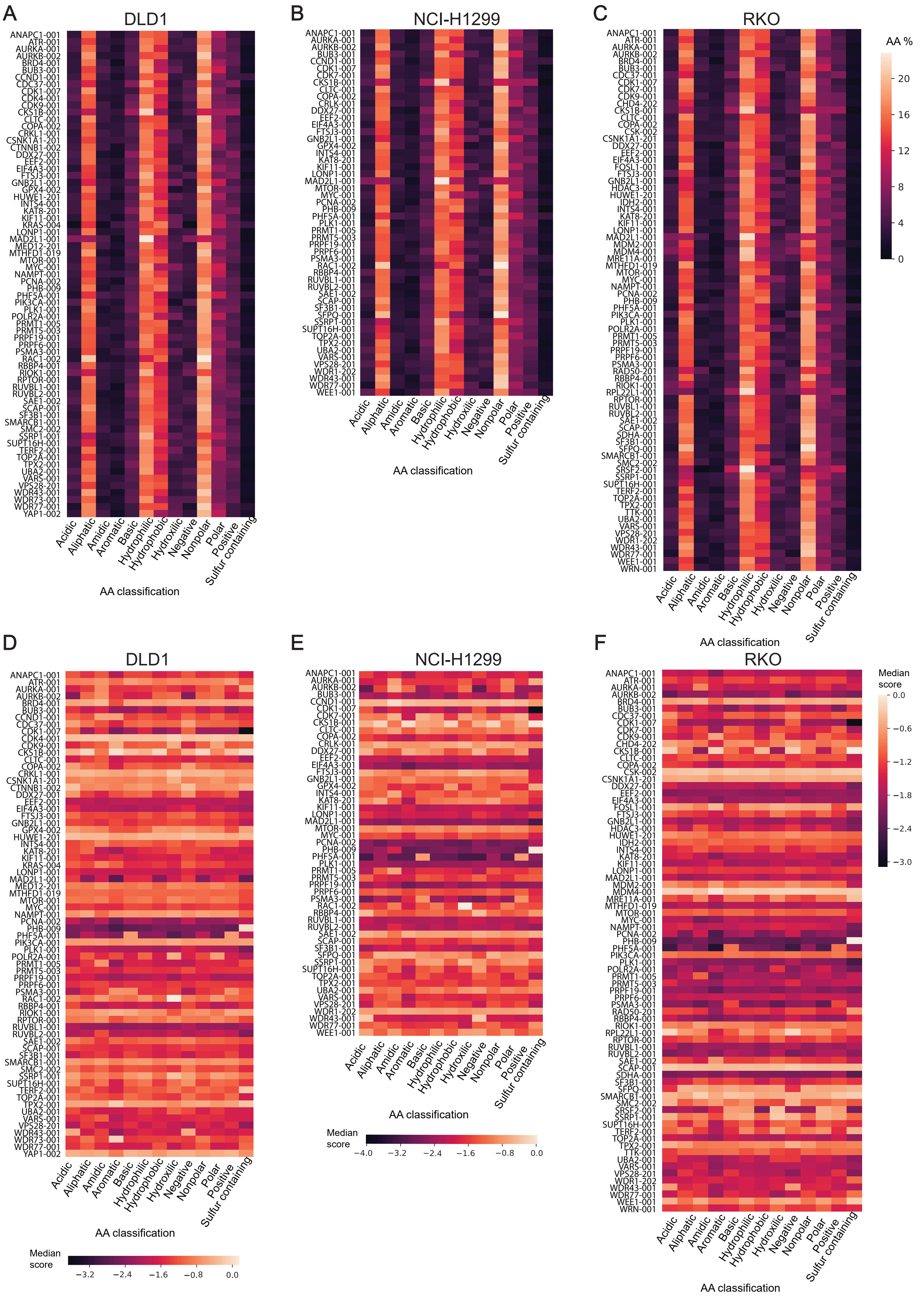
A
DLD1
ANAPC1-001
ATR-001
AURKA-001
AURKB-002
BRD4-001
BUB3-001
CCND1-001
CDC37-001
CDK1-007
CDK4-001
CDK9-001
CKS1B-001
CLTC-001
COPA-002
CRKL1-001
CSNK1A1-201
CTNNB1-002
DDX27-001
EEF2-001
EIF4A3-001
FTSJ3-001
GNB2L1-001
GPX4-002
HUWE1-201
INTS4-001
KAT8-201
KIF11-001
KRAS-004
LONP1-001
MAD2L1-001
MED12-201
MTHFD1-019
MTOR-001
MYC-001
NAMPT-001
PCNA-002
PHB-009
PHF5A-001
PIK3CA-001
PLK1-001
POLR2A-001
PRMT1-005
PRMT5-003
PRPF19-001
PRPF6-001
PSMA3-001
RAC1-002
RBBP4-001
RIOK1-001
RPTOR-001
RUVBL1-001
RUVBL2-001
SAE1-002
SCAP-001
SF3B1-001
SMARCB1-001
SMC2-002
SSRP1-001
SUPT16H-001
TERF2-001
TOP2A-001
TPX2-001
UBA2-001
VARS-001
VPS28-201
WDR43-001
WDR73-001
WDR77-001
YAP1-002
Acidic
Aliphatic
Amidic
Aromatic
Basic
Hydrophilic
Hydrophobic
Hydroxilic
Negative
Nonpolar
Polar
Positive
Sulfur containing
AA classification
B
NCI-H1299
ANAPC1-001
AURKA-001
AURKB-002
BUB3-001
CCND1-001
CDK1-007
CDK7-001
CKS1B-001
CLTC-001
COPA-002
DDX27-001
EEF2-001
EIF4A3-001
FTSJ3-001
GNB2L1-001
GPX4-002
INTS4-001
KAT8-201
KIF11-001
LONP1-001
MAD2L1-001
MTOR-001
MYC-001
PCNA-002
PHB-009
PHF5A-001
PLK1-001
PRMT1-005
PRMT5-003
PRPF19-001
PRPF6-001
PSMA3-001
RAC1-002
RBBP4-001
RUVBL1-001
RUVBL2-001
SAE1-002
SCAP-001
SF3B1-001
SFPQ-001
SSRP1-001
SUPT16H-001
TOP2A-001
TPX2-001
UBA2-001
VARS-001
VPS28-201
WDR1-202
WDR43-001
WDR77-001
WEE1-001
Acidic
Aliphatic
Amidic
Aromatic
Basic
Hydrophilic
Hydrophobic
Hydroxilic
Negative
Nonpolar
Polar
Positive
Sulfur containing
AA classification
C
RKO
ANAPC1-001
ATR-001
AURKA-001
AURKB-002
BRD4-001
BUB3-001
CDC37-001
CDK1-007
CDK7-001
CDK9-001
CHD4-202
CKS1B-001
CLTC-001
COPA-002
CSK-002
CSNK1A1-201
DDX27-001
EEF2-001
EIF4A3-001
FOSL1-001
FTSJ3-001
GNB2L1-001
HDAC3-001
HUWE1-201
IDH2-001
INTS4-001
KAT8-201
KIF11-001
LONP1-001
MAD2L1-001
MDM2-001
MDM4-001
MRE11A-001
MTHFD1-019
MTOR-001
MYC-001
NAMPT-001
PCNA-002
PHB-009
PHF5A-001
PIK3CA-001
PLK1-001
POLR2A-001
PRMT1-005
PRMT5-003
PRPF19-001
PRPF6-001
PSMA3-001
RAD50-201
RBBP4-001
RIOK1-001
RPL22L1-001
RPTOR-001
RUVBL1-001
RUVBL2-001
SAE1-002
SCAP-001
SDHA-001
SF3B1-001
SFPQ-001
SMARCB1-001
SMC2-002
SRSF2-001
SSRP1-001
SUPT16H-001
TERF2-001
TOP2A-001
TPX2-001
TTK-001
UBA2-001
VARS-001
VPS28-201
WDR1-202
WDR43-001
WDR77-001
WEE1-001
WRN-001
Acidic
Aliphatic
Amidic
Aromatic
Basic
Hydrophilic
Hydrophobic
Hydroxilic
Negative
Nonpolar
Polar
Positive
Sulfur containing
AA classification
AA %
20
16
12
8
4
0
D
DLD1
AA classification
Median score
−3.2
−2.4
−1.6
−0.8
0.0
E
NCI-H1299
CRLK-001
AA classification
Median score
−4.0
−3.2
−2.4
−1.6
−0.8
0.0
F
RKO
AA classification
Median score
0.0
−0.6
−1.2
−1.8
−2.4
−3.0

Figure S4 Correlation of AA class annotation to functional score.

A-C) Percentage of amino acids in each amino acid class per transcript (see figure **S3I** for class assignments). (**A**) DLD1 cell line, (**B**) NCI-H1299 cell line, (**C**) RKO cell line. **D-F)** Median z-score for each amino acid class per transcript (**D**) DLD1 cell line, (**E**) NCI-H1299 cell line, (**F**) RKO cell line.

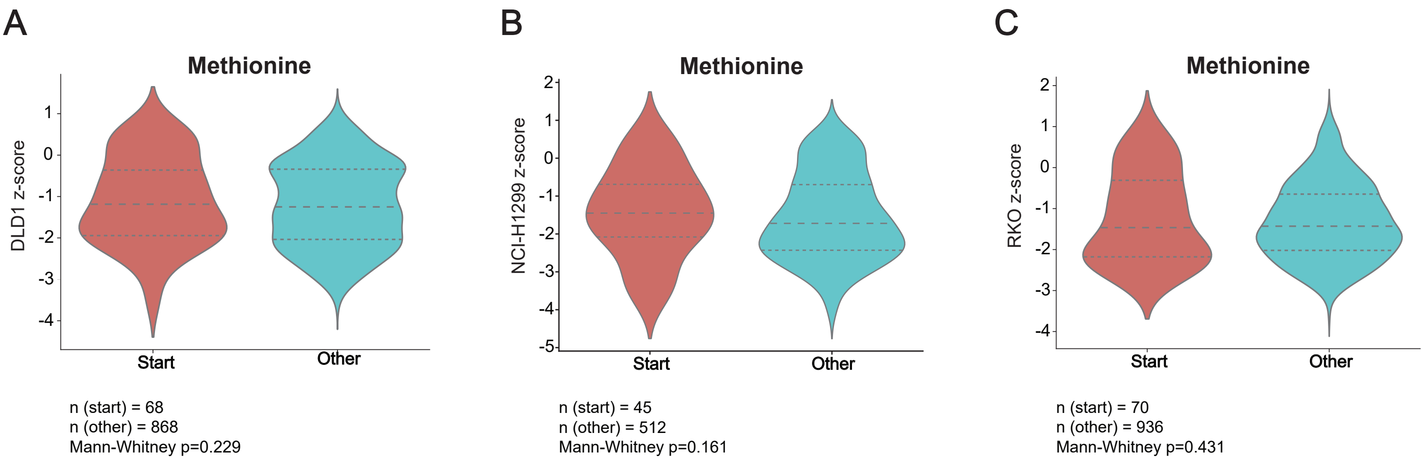

Figure S5 Distribution of z-scores for guide RNAs targeting the amino acid methionine at the start or other position in the protein.
Results shown for: **A)** DLD1 cell line, **B)** NCI-H1299 cell line, **C)** RKO cell line.

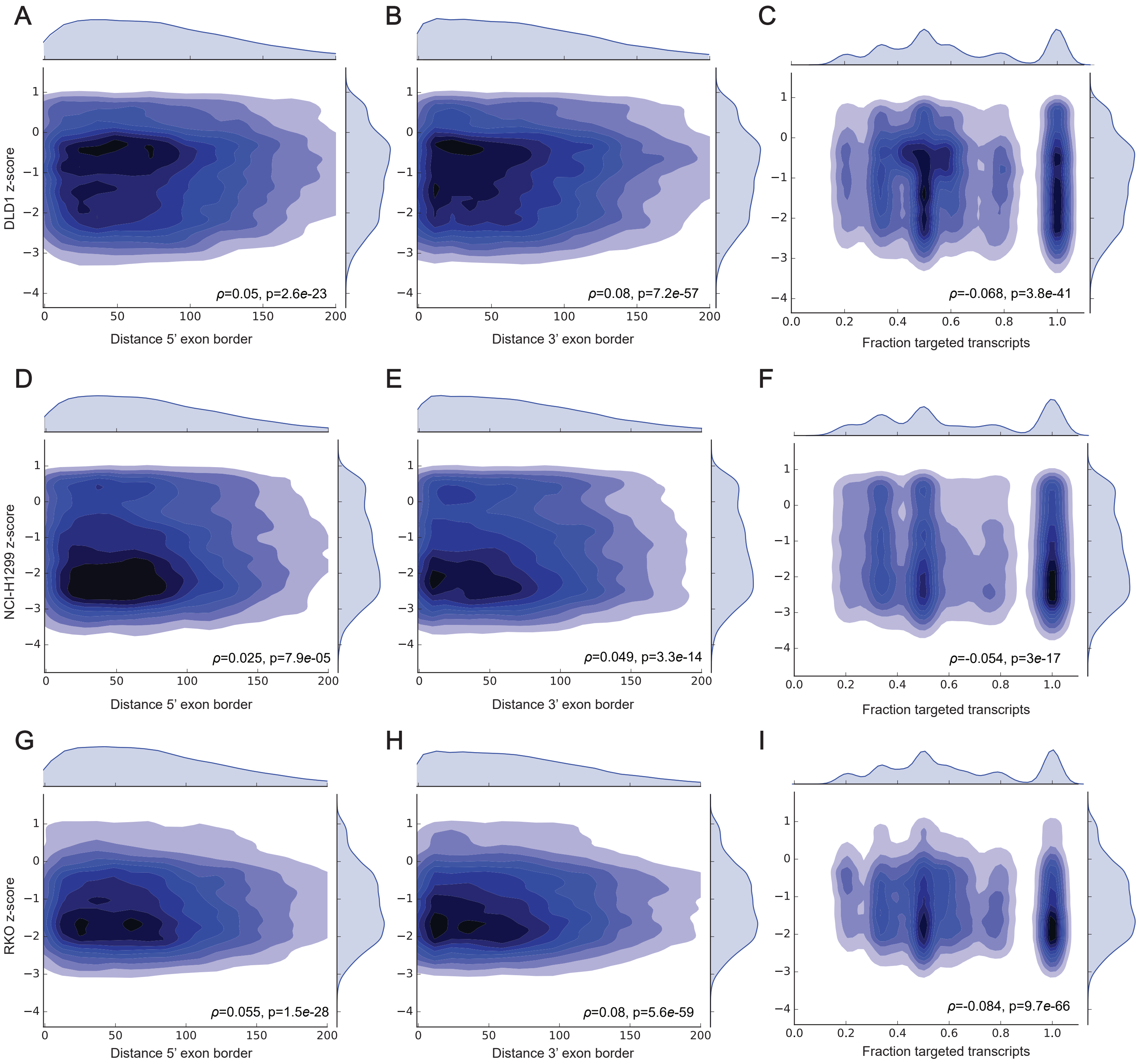

Figure S6 Correlation of transcript level annotations to functional scores.

A) Density plot showing the relation between DLD1 z-score and the distance of the guide RNA predicted cleavage site to the 5' exon border. **B)** Density plot showing the relation between DLD1 z-score and the distance of the guide RNA predicted cleavage site to the 3' exon border. **C)** Density plot showing the relation between DLD1 z-score and the fraction of targeted transcripts of a gene by a guide RNA. **D)** Density plot showing the relation between NCI-H1299 z-score and the distance of the guide RNA predicted cleavage site to the 5' exon border. **E)** Density plot showing the relation between NCI-H1299 z-score and the distance of the guide RNA predicted cleavage site to the 3' exon border. **F)** Density plot showing the relation between NCI-H1299 z-score and the fraction of targeted transcripts of a gene by a guide RNA. **G)** Density plot showing the relation between RKO z-score and the distance of the guide RNA predicted cleavage site to the 5' exon border. **H)** Density plot showing the relation between RKO z-score and the distance of the guide RNA predicted cleavage site to the 3' exon border. **I)** Density plot showing the relation between RKO z-score and the fraction of targeted transcripts of a gene by a guide RNA.

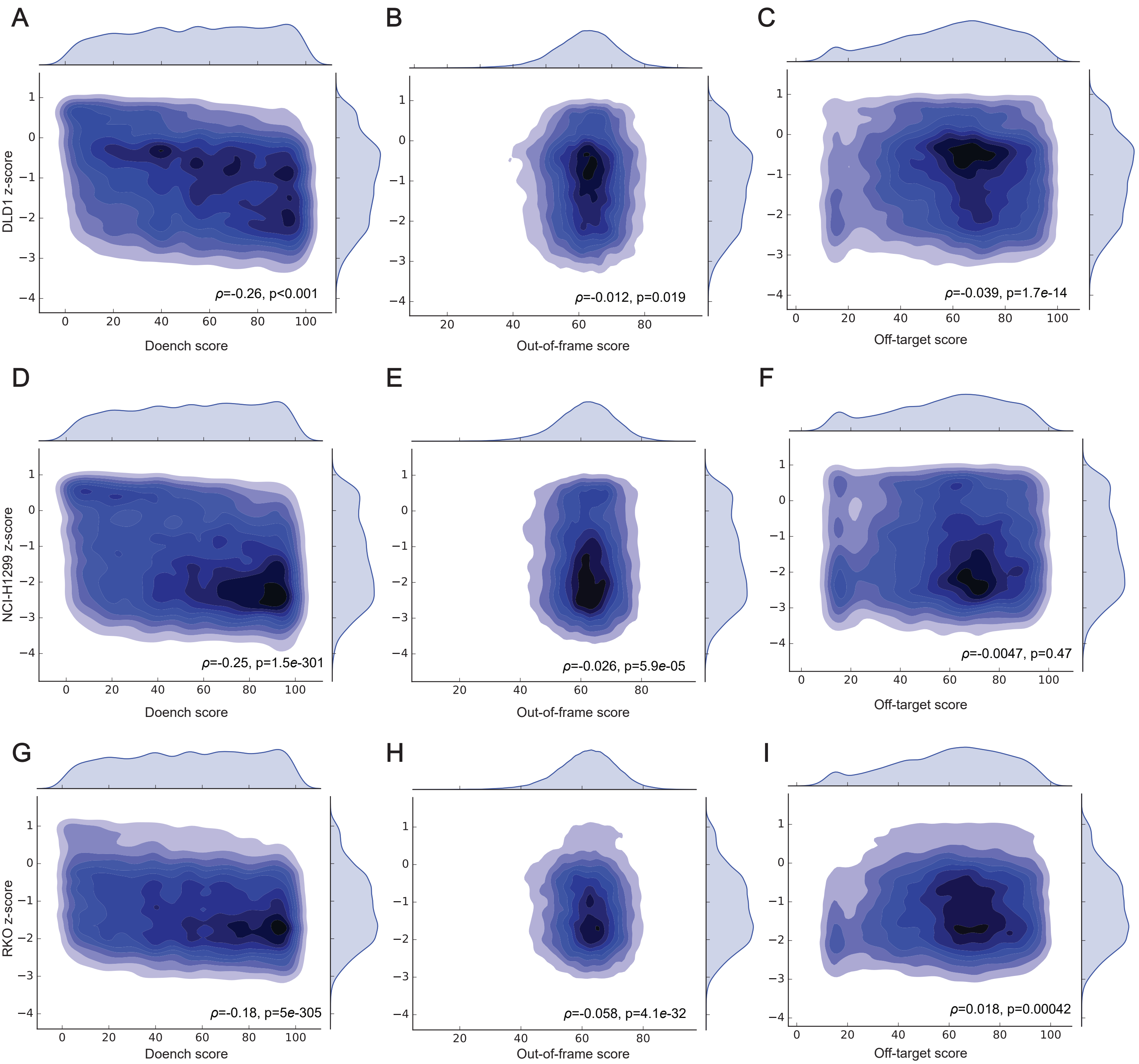

Figure S7 Correlation of nucleotide level annotations to functional scores.
A) Density plot showing the relation between DLD1 z-score and the Doench score. **B)** Density plot showing the relation between DLD1 z-score and the out-of-frame score. **C)** Density plot showing the relation between DLD1 z-score and the off-target score of a guide RNA. **D)** Density plot showing the relation between NCI-H1299 z-score and the Doench score. **E)** Density plot showing the relation between NCI-H1299 z-score and the out-of-frame score. **F)** Density plot showing the relation between NCI-H1299 z-score and the off-target score of a guide RNA. **G)** Density plot showing the relation between RKO z-score and the Doench score. **H)** Density plot showing the relation between RKO z-score and the out-of-frame score. **I)** Density plot showing the relation between RKO z-score and the off-target score of a guide RNA.

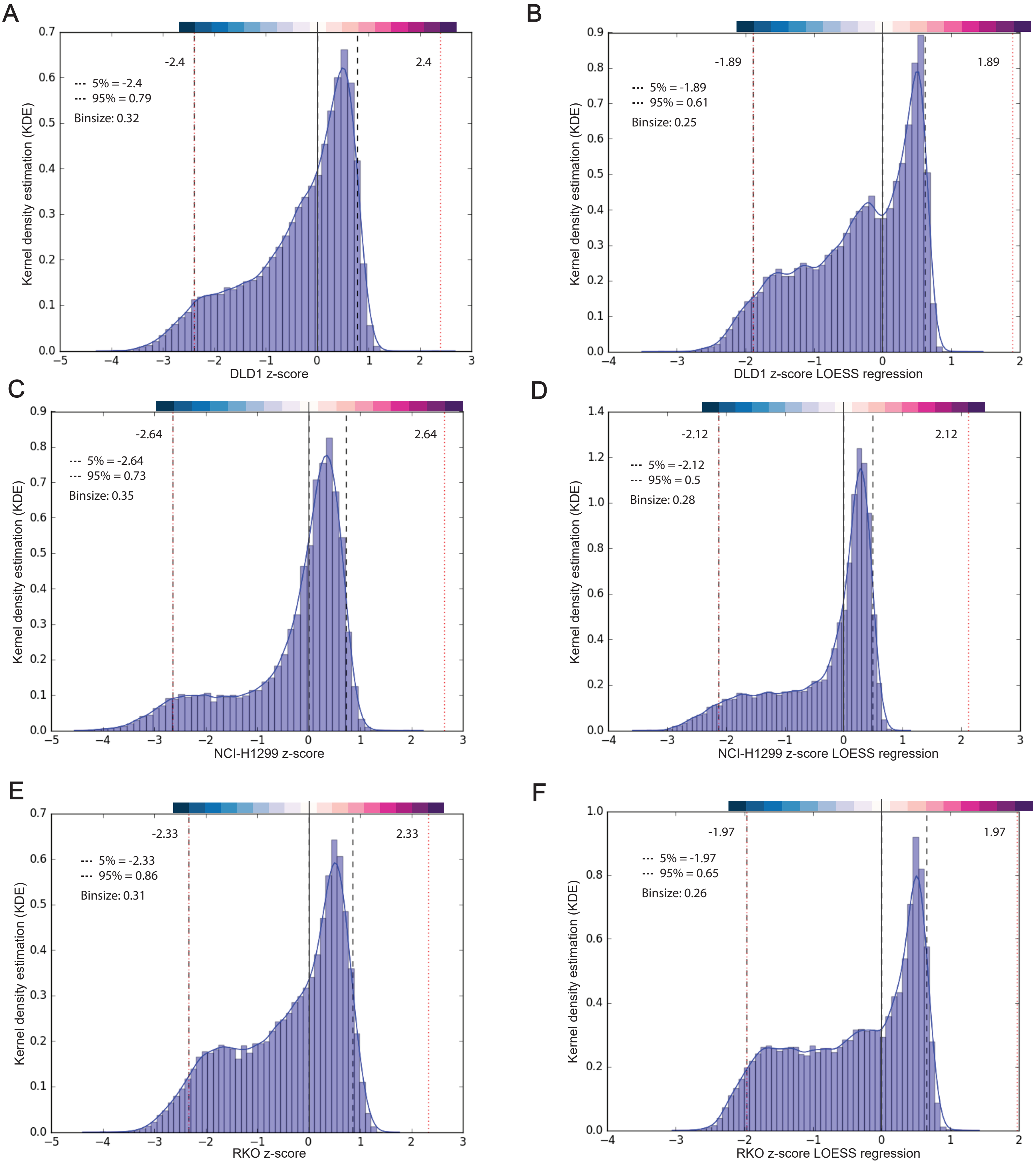

Figure S8 Score distributions with color legend.

The CRISPRO color legend used here consists of 17 bins, from blue to dark purple, centered on 0. CRISPRO determines bin size and boundaries for each functional CRISPR score, over all the proteins in the dataset. Either the 5th or 95th percentile (and its inverse) of the score distribution, whichever is farther from 0, is set as the upper and lower border of the outermost bins. Every score lower or higher than this value will fall into those outer bins. Outer bin borders are presented by red dotted line. The rest of the bins are evenly sized between the borders, resulting in a scale centered on 0. **A)** DLD1 z-score. **B)** DLD1 z-score LOESS regression. **C)** NCI-H1299 z-score. **D)** NCI-H1299 z-score LOESS regression. **E)** RKO z-score. **F)** RKO z-score LOESS regression.

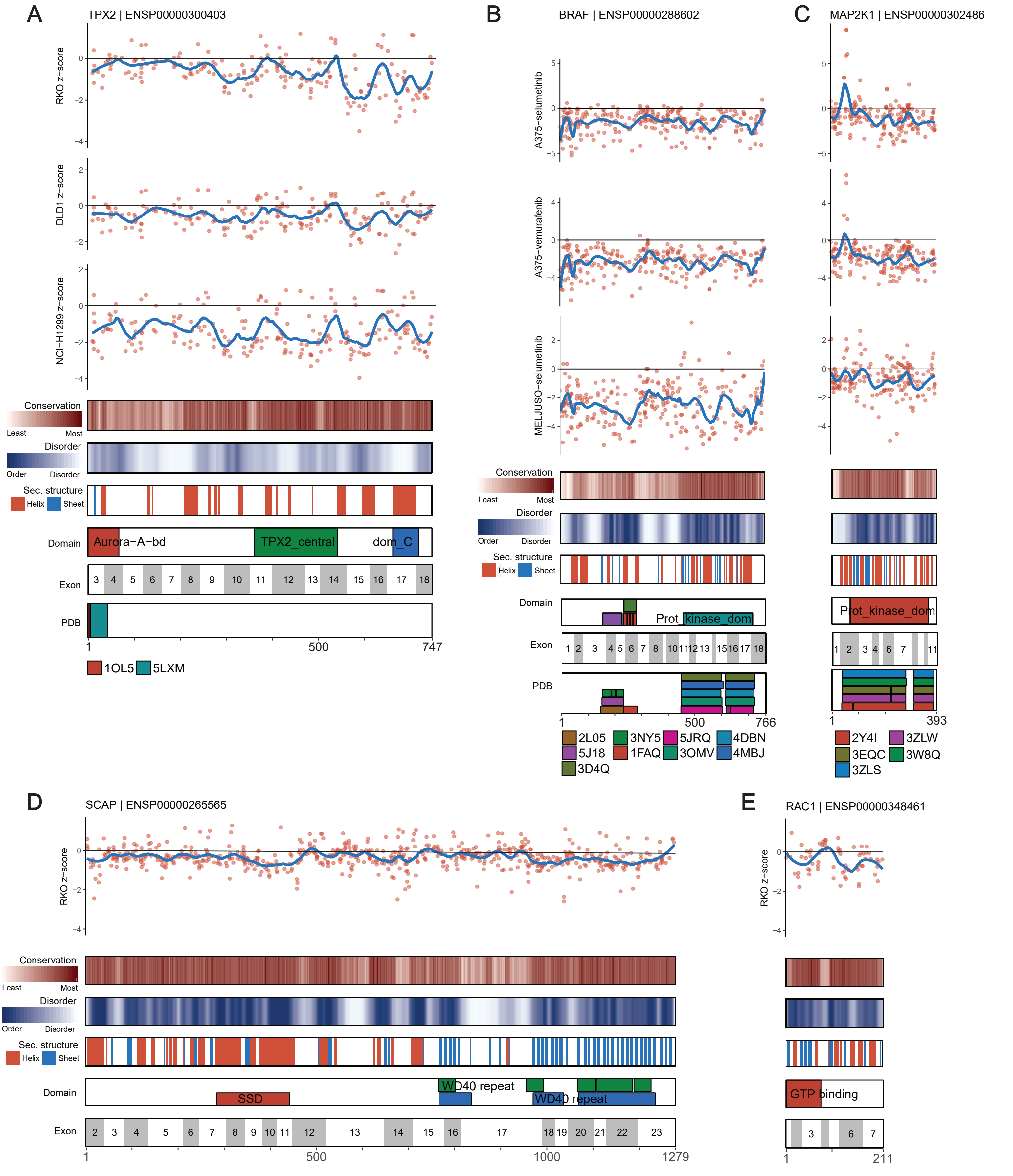

Figure S9 CRISPRO linear maps.

A) CRISPRO linear map of TPX2. Data from Munoz *et al.* [7] From top to bottom: Graph showing RKO z-scores (each guide RNA with corresponding score, mapped to protein sequence (red dot), and LOESS interpolation (blue line)). DLD1 z-scores. NCI-H1299 z-scores. Predicted conservation scores (PROVEAN). Predicted disorder scores. Predicted secondary structure consensus. Domain annotations (InterPro). Exon annotations. Initial PDB alignments. **B-C)** CRISPRO linear maps from non-genome editing experimental data. Ectopic saturation mutagenesis data from Brenan *et al.* [32] of BRAF **(B)** and MAP2K1 **(C)**. From top to bottom: Graph showing A375 with selumetinib scores (each guide RNA with corresponding score, mapped to protein sequence (red dot), and LOESS interpolation (blue line)). A375 with vemurafenib scores. MELJUSO with selumetinib scores. Predicted conservation scores (PROVEAN). Predicted disorder scores. Predicted secondary structure consensus. Domain annotations (InterPro). Exon annotations. Initial PDB alignments. **D-E)** CRISPRO linear maps of genes uniquely called as hits by Munoz *et al.* [7] or CRISPRO (also see Figure S1). SCAP was called as a hit-gene by CRISPRO but not by Munoz *et al*. and RAC1 was called as a hit gene by Munoz *et al.* but not by CRISPRO. From top to bottom: Graph showing RKO z-scores (each guide RNA with corresponding score, mapped to protein sequence (red dot), and LOESS interpolation (blue line)). Predicted conservation scores (PROVEAN). Predicted disorder scores. Predicted secondary structure consensus. Domain annotations (InterPro). Exon annotations.

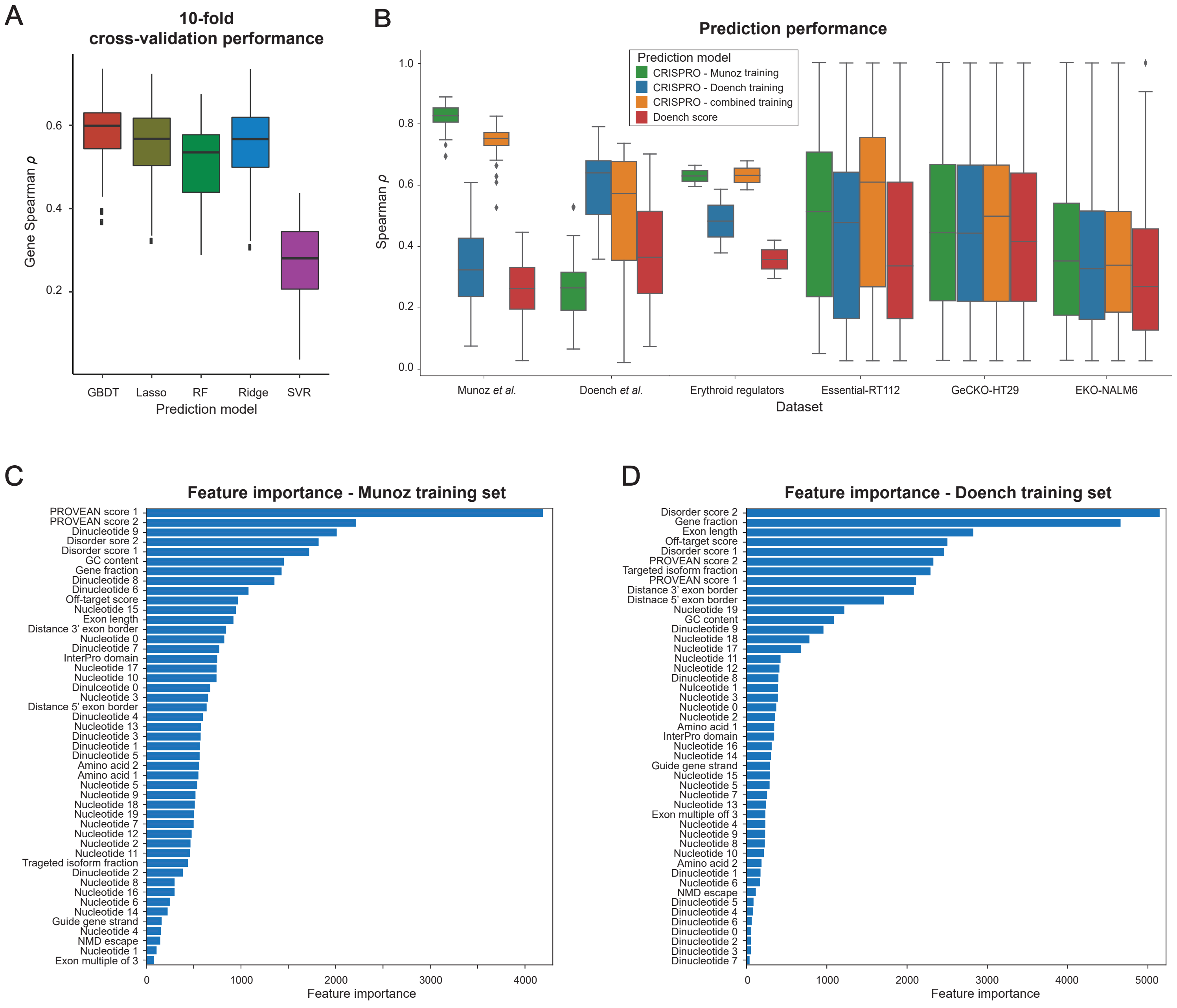

Figure S10 Prediction of functional CRISPR scores.

A) Spearman correlation of predicted sgRNA scores obtained via 10-fold cross-validation and observed CRISPR sgRNA scores from the Munoz *et al*. dataset for indicated prediction models. **B)** Spearman correlation of predicted and observed CRISPR sgRNA scores from all datasets described in this paper stratified by dataset used to train the model. Combined training indicates the CRISPRO prediction model trained on both the Munoz *et al.* and Doench *et al.* datasets. **C)** Feature importance in GBDT model trained on Munoz *et al*. dataset by information gain when a feature is used to split the Munoz *et al*. training data. Positional nucleotide features are 0-indexed (i.e. nucleotide 0 is in position 1 of the spacer sequence, dinucleotide 0 corresponds to positions 1 and 2 of spacer, where position 20 is PAM proximal). **D)** Feature importance in GBDT model trained on Doench *et al*. dataset by information gain when a feature is used to split the Doench et al. training data.

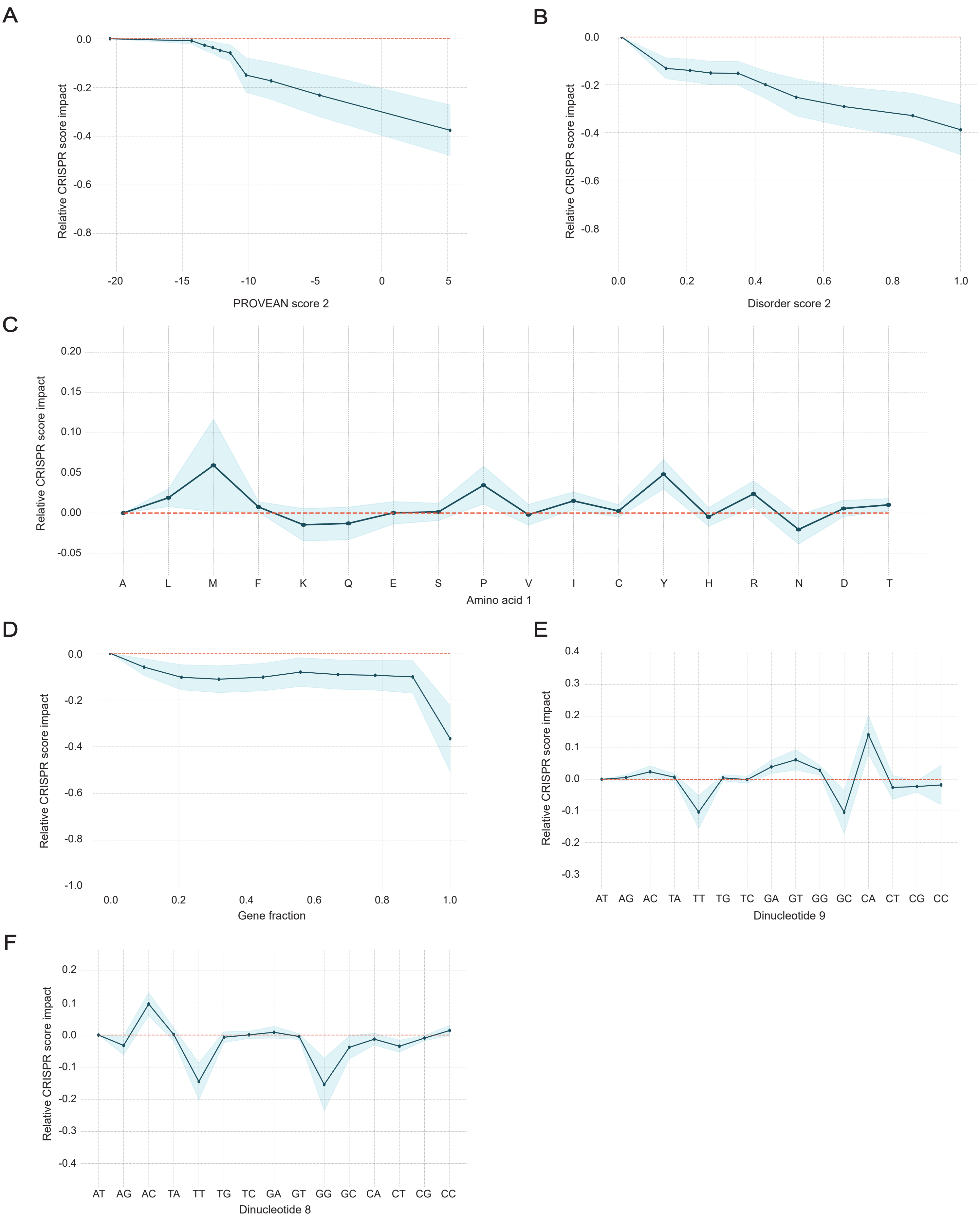

Figure S11 Partial dependency plot of features in CRISPRO prediction.
Blue fill shows standard deviation of relative CRISPR score impact for each input score: **A)** PROVEAN score 2; **B)** Disorder score 2; **C)** Amino acid 1; **D)** Gene fraction; **E)** Dinucleotide 9; **F)** Dinucleotide 8. Higher relative CRISPR score impact can be interpreted as higher loss of function capability. For amino acid 1, relative CRISPR score impact is not shown for amino acids G and W are not shown due to one hot encoding and recursive feature elimination. For dinucleotides, AA is not shown in the model due to one hot encoding and recursive feature elimination.

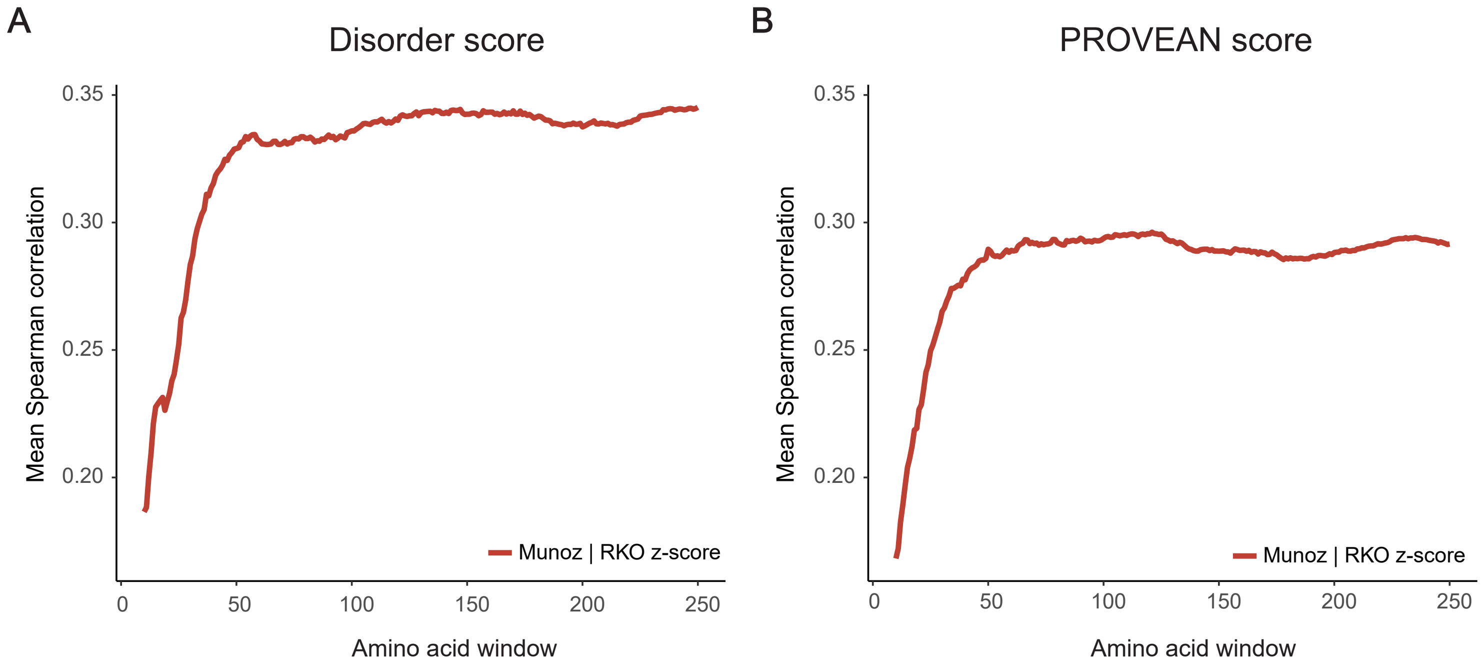

Figure S12 LOESS regression span parameter.
A) Correlation of LOESS regression CRISPR scores and corresponding positional disorder scores as a function of the span parameter in LOESS regression. Span parameter defined as amino acid window / protein length (AA/L). **B)** Correlation of LOESS regression CRISPR scores and corresponding positional PROVEAN scores a function of the span parameter in LOESS regression.
